# The biomarker and causal roles of homoarginine in the development of cardiometabolic diseases: an observational and Mendelian randomization analysis

**DOI:** 10.1038/s41598-017-01274-6

**Published:** 2017-04-25

**Authors:** Ilkka Seppälä, Niku Oksala, Antti Jula, Antti J. Kangas, Pasi Soininen, Nina Hutri-Kähönen, Winfried März, Andreas Meinitzer, Markus Juonala, Mika Kähönen, Olli T. Raitakari, Terho Lehtimäki

**Affiliations:** 1 Department of Clinical Chemistry, Fimlab Laboratories, Faculty of Medicine and Life Sciences, University of Tampere, Tampere, Finland; 2 Division of Vascular Surgery, Department of Surgery, Tampere University Hospital, Tampere, Finland; 3 Health and Functional Capacity, National Institute for Health and Welfare, Turku, Finland; 4 Computational Medicine, Faculty of Medicine, University of Oulu, Oulu, Finland; 5 NMR Metabolomics Laboratory, School of Pharmacy, University of Eastern Finland, Kuopio, Finland; 6 Department of Paediatrics, University of Tampere and Tampere University Hospital, Tampere, Finland; 7 Institute of Public Health, Social and Preventive Medicine, Mannheim Medical Faculty, University of Heidelberg, Mannheim, Germany; 8 Synlab Services GmbH, Mannheim, Germany; 9 Clinical Institute of Medical and Chemical Laboratory Diagnostics, Medical University of Graz, Graz, Austria; 10 Research Centre of Applied and Preventive Cardiovascular Medicine, University of Turku, Turku, Finland; 11 Department of Medicine, Turku University Hospital and University of Turku, Turku, Finland; 12 Department of Clinical Physiology, Tampere University Hospital, and Faculty of Medicine and Life Sciences, University of Tampere, Tampere, Finland; 13 Department of Clinical Physiology and Nuclear Medicine, Turku University Hospital, Turku, Finland

## Abstract

High L-homoarginine (hArg) levels are directly associated with several risk factors for cardiometabolic diseases whereas low levels predict increased mortality in prospective studies. The biomarker role of hArg in young adults remains unknown. To study the predictive value of hArg in the development of cardiometabolic risk factors and diseases, we utilized data on high-pressure liquid chromatography-measured hArg, cardiovascular risk factors, ultrasound markers of preclinical atherosclerosis and type 2 diabetes from the population-based Young Finns Study involving 2,106 young adults (54.6% females, aged 24–39). We used a Mendelian randomization approach involving tens to hundreds of thousands of individuals to test causal associations. In our 10-year follow-up analysis, hArg served as an independent predictor for future hyperglycaemia (OR 1.31, 95% CI 1.06–1.63) and abdominal obesity (OR 1.60, 95% 1.14–2.30) in men and type 2 diabetes in women (OR 1.55, 95% CI 1.02–2.41). The MR analysis revealed no evidence of causal associations between serum hArg and any of the studied cardiometabolic outcomes. In conclusion, lifetime exposure to higher levels of circulating hArg does not seem to alter cardiometabolic disease risk. Whether hArg could be used as a biomarker for identification of individuals at risk developing cardiometabolic abnormalities merits further investigation.

## Introduction

Accumulating evidence indicates that low levels of serum non-proteinogenic amino acid L-homoarginine (hArg) are associated with an increased risk of death from cardiovascular diseases, including heart failure, sudden cardiac death and fatal strokes, in various patient populations^1–6^. The association of hArg with adverse cardiovascular outcomes also appears to hold true in a population-based cohort of older adults^7^ as well as a multi-ethnic United States population^8^. On the other hand, in cross-sectional analyses of these population samples, hArg was positively associated with several cardiovascular risk factors including hypertension, obesity, dysglycaemia, insulin resistance and dyslipidaemia, referring to the possibility that hArg could also play a causal role in the development of cardiometabolic diseases. Interestingly, recent experimental data indicates that hArg promotes the calcification of vascular smooth muscle cells^9^ and may thus indeed have a causal role in the pathogenesis of atherosclerosis.

Supplementation with L-arginine (Arg), the primary precursor of nitric oxide (NO), unexpectedly increased cardiovascular events and mortality in a small randomized controlled trial (RCT) in patients with acute coronary syndrome^10^. Whether hArg administration in the secondary prevention setting would have the same detrimental effects as Arg, or whether increased levels of hArg would reduce the rate of cardiovascular events and mortality as suggested by most of the prospective studies, is currently not known. However, before any clinical trials using hArg supplementation or a pharmacological intervention to modify circulating hArg levels, it is rational to conduct an epidemiological investigation of its causality and potential pleiotropic effects, i.e. both adverse and/or beneficial effects, by using genetically determined hArg to avoid inaccurate conclusions due to the reverse causation and residual confounding related to all observational studies. Moreover, because common cardiometabolic diseases such as type 2 diabetes mellitus (T2DM) and coronary artery disease (CAD) develop over several decades during one’s lifespan before any symptoms or clinical manifestations, any clinical trials on the effects of the lifetime exposure to high hArg in primary prevention settings are not feasible in practice.

So far, no prospective studies have been conducted to study the predictive value of circulating hArg levels on the development of metabolic abnormalities in early adulthood. As metabolic syndrome (Mets) or a high body mass index (BMI) are strong predictors of future T2DM and cardiovascular disease manifestations^11, 12^, we investigated whether hArg levels could predict the incidence of Mets components or obesity (BMI >30 kg/m^2^) and high insulin as well as incident T2DM and preclinical atherosclerosis in a population-based prospective cohort of young adults without clinical cardiovascular diseases. Moreover, we applied quantitative serum nuclear magnetic resonance (NMR) metabolomics to study the detailed molecular associations with hArg in young men and women. Furthermore, although we have individual-level genome-wide genetic data available for the Cardiovascular Risk in Young Finns Study (YFS) participants, we used summary-level data from several large-scale meta-analyses of genome-wide association studies involving tens to hundreds of thousands of individuals available in the public domain to increase the statistical power to detect potentially causal associations between hArg and metabolites, cardiometabolic risk factors, T2DM and CAD.

## Results

**Figure 1:**
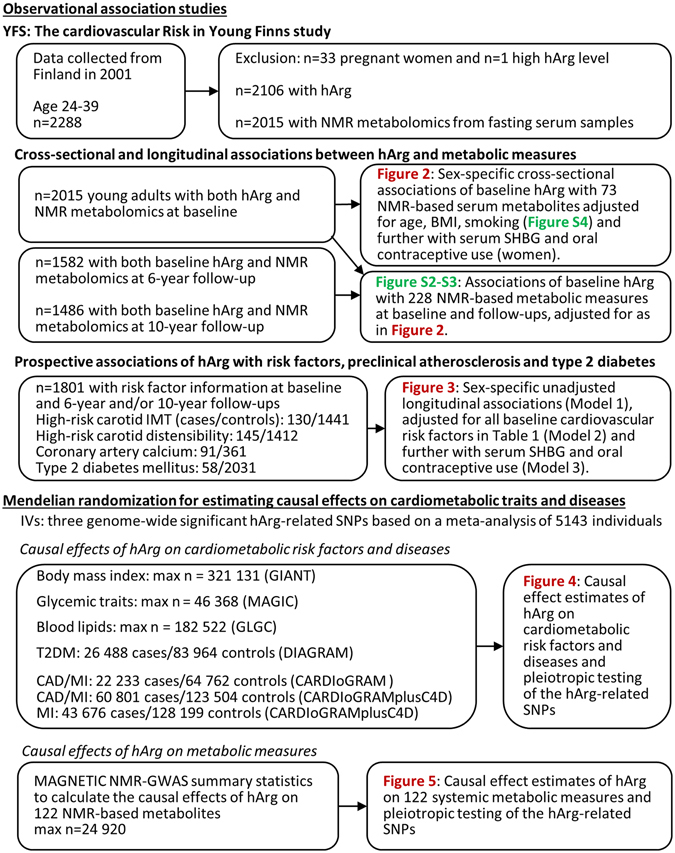
Overview of the study design, data sources and statistical analyses.

An overview of the study flow, data sources and statistical analyses is illustrated in Fig. 1.

### Clinical characteristics and cross-sectional determinants of serum hArg levels in YFS

Baseline characteristics of the YFS study subjects are presented in Table 1. To examine the determinants of hArg levels at baseline (2001), we performed a multivariate linear regression analysis using a bi-directional step-wise procedure. When all cardiometabolic risk factor variables shown in Table 1, along with serum SHBG, were included in the same stepwise multivariable linear regression model, hArg was independently and positively associated with male sex, BMI, SHBG, triglycerides and LDL cholesterol (Table 2). Inverse associations of hArg were observed with daily smoking and age. The overall R^2^ for hArg was 0.114, suggesting that 11.4% of the variation in hArg concentrations was explained by the selected variables. When the three hArg-related SNPs were further added to the model selection procedure, the overall R^2^ statistic increased to a value of 22.4% (n = 1895) while the significance of the other explanatory variables remained roughly the same. Prompted by the association with SHBG and a detailed metabolic profiling, the association of hArg with different hormonal contraceptive methods in women is shown in Figure S1. In women, HArg was positively associated with the use of combined oral contraceptives (containing oestrogen), but not with the use of progestin-only contraceptives (Figure S1).

**Table 1:** Baseline descriptive data for the YFS cohort in 2001.

	All	Men	Women
Number of subjects (%)	2106	957 (45.4)	1149 (54.6)
Age (years)	31.7 (5.0)	31.6 (5.0)	31.7 (5.0)
Homoarginine (hArg) (µmol/L)	1.85 (0.65)	1.93 (0.61)	1.79 (0.68)
*ln*hArg (µmol/L)	0.56 (0.34)	0.61 (0.30)	0.52 (0.36)
LDL cholesterol (mmol/L)	3.27 (0.84)	3.42 (0.90)	3.14 (0.77)
HDL cholesterol (mmol/L)	1.29 (0.31)	1.17 (0.27)	1.39 (0.30)
Triglycerides (mmol/L)	1.26 (0.64)	1.41 (0.70)	1.13 (0.55)
Systolic blood pressure (mmHg)	117 (13)	121 (12)	113 (12)
Diastolic blood pressure (mmHg)	72 (11)	73 (11)	69 (10)
C-reactive protein (mg/L)	1.9 (4.0)	1.5 (3.4)	2.1 (4.4)
Glucose (mmol/L)	5.1 (0.85)	5.2 (0.93)	4.9 (0.75)
Insulin (IU/L)	7.7 (5.7)	7.6 (5.8)	7.8 (5.7)
Body mass index (kg/m^2^)	25.0 (4.4)	25.6 (4.1)	24.4 (4.5)
Waist circumference (cm)	84 (12)	90 (11)	79 (11)
Daily smokers (%)	520 (24.7)	288 (30.1)	232 (20.2)
Family history of CAD (%)	281 (13.3)	123 (12.9)	158 (13.8)

Statistics are mean (SD) or n (%); *ln*hArg is natural log-transformed. CAD, coronary artery disease; LDL, low-density lipoprotein; HDL, high-density lipoprotein.

**Table 2:** Cross-sectional stepwise multivariable linear regression modelling for homoarginine (hArg) (n = 2057).

Explanatory variable	β	95% CI	P-value
Male sex	0.14	[0.10, 0.17]	4.1 × 10^−13^
Body mass index (kg/m^2^)	0.014	[0.0093, 0.018]	1.2 × 10^−9^
Daily smoking	−0.089	[−0.12, −0.056]	1.3 × 10^−7^
Age (years)	−0.0067	[−0.0097, −0.0038]	7.8 × 10^−6^
*ln*SHBG (nmol/L)	0.061	[0.032, 0.089]	3.2 × 10^−5^
*ln*Triglycerides (mmol/L)	0.045	[0.019, 0.072]	8.2 × 10^−4^
LDL cholesterol (mmol/L)	0.022	[0.0045, 0.040]	0.014
*ln*CRP (mg/L)	0.016	[0.0023, 0.029]	0.021

**Statistics:** In the bi-directional stepwise regression modelling applied, serum hArg was used as a dependent variable and all the variables shown in Table 1 and *ln*SHBG as explanatory variables. Those variables that were selected by Akaike’s information criterion (AIC) using the stepAIC R function with the default settings and had a p-value < 0.05 are shown above. HArg, SHBG, triglycerides and CRP were natural log-transformed. For the continuous variables, β (95% CI) are shown for each 1‐unit change in the variable. **Abbreviations:** SHBG, sex hormone-binding globulin; LDL, low-density lipoprotein; CRP, C-reactive protein.

### Longitudinal and cross-sectional associations of hArg with 228 serum metabolites in YFS

**Figure 2:**
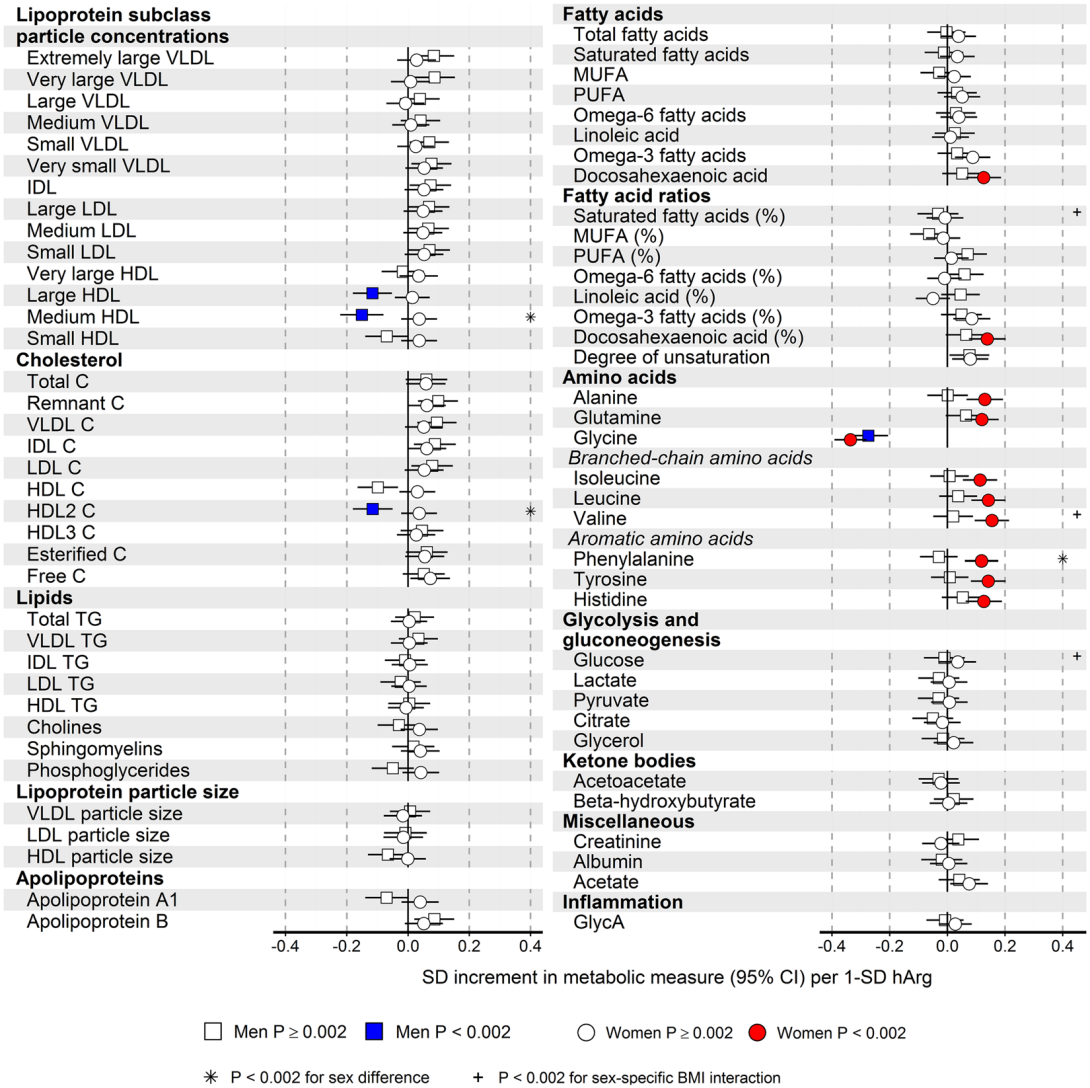
Sex-specific cross-sectional associations of baseline hArg with 73 NMR-based serum metabolites, adjusted for age, body mass index (BMI), daily smoking, serum SHBG and oral contraceptive use (in women). The analyses were conducted for 867 men and 1097 women. Squares indicate men, and circles represent women. Open and closed symbols indicate *P* ≥ 0.002 and *P* < 0.002, respectively. Sex differences with *P* < 0.002 are marked by asterisks. BMI interactions with *P* < 0.002 are marked by the plus or minus sign, depending on the direction of the estimated interaction effect.

The cross-sectional and longitudinal (6- to 10-year follow-up) associations of baseline hArg with 228 serum metabolites are shown in Figures S2–S3. For both sexes, the strong negative association of glycine with hArg persisted over time during 10 years of follow-up. For all subjects, glycine, histidine and tyrosine showed consistent associations with hArg at each time point (Figure S3). The cross-sectional associations of hArg with 73 selected metabolomics measures adjusted for age, BMI and daily smoking are illustrated separately for men and women in Figure S4. In women, more metabolic measures were associated with hArg compared to men, i.e. 33 measures for women and 9 for men (P < 0.002). In women, hArg was positively associated with the smallest very-low-density lipoprotein (VLDL), small and medium HDL, the triglyceride content of different lipoprotein classes, as well as fatty acids, several amino acids and glycoprotein acetyls (GlycA), which is a novel marker of inflammation (Figure S4). Indeed, when further adjusting for oral contraceptive use (in women) and serum SHBG (in both sexes), these associations were largely reduced to non-significant, except for the positive associations of amino acids and docosahexaenoic acid with hArg in women (Fig. 2). For both sexes, glycine showed a strong negative association with hArg, whereas the negative association of HDL subclasses and hArg was seen only in men (Fig. 2). Significant men-specific interactions were observed between hArg and BMI on glucose, valine and saturated fatty acids (%) (Fig. 2).

### Longitudinal relations of hArg with incident obesity, Mets components, preclinical atherosclerosis and T2DM in YFS

**Figure 3:**
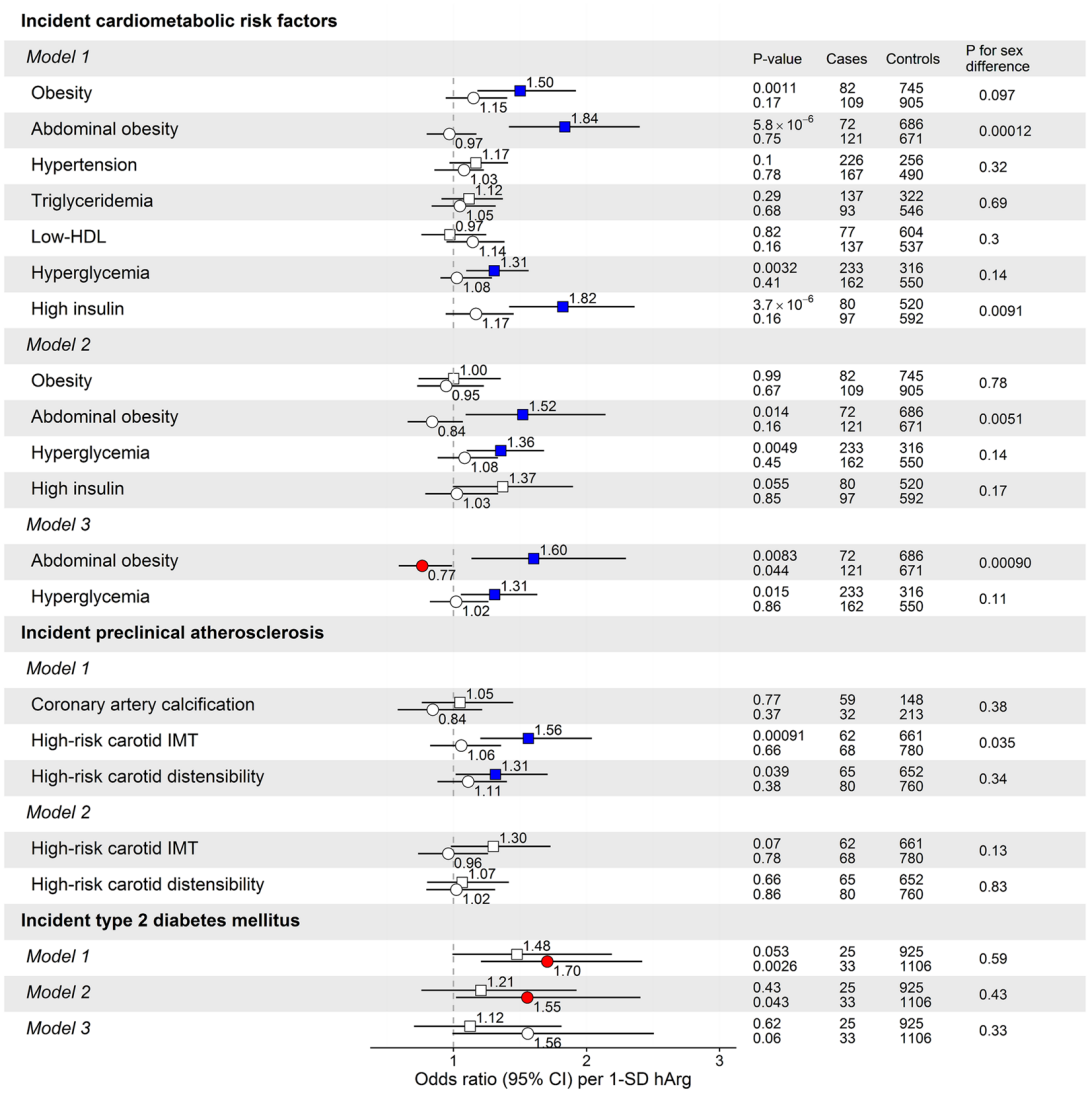
Sex-specific prospective (observational) associations of baseline hArg (in 2001) and cardiometabolic risk factors, preclinical atherosclerosis and type 2 diabetes mellitus (T2DM) during a 10-year follow-up in YFS. The prospective associations are shown as unadjusted (Model 1), adjusted for all baseline cardiometabolic risk factors shown in Table 1 (Model 2), and further adjusted for baseline serum steroid hormone binding globulin (SHBG) and oral contraceptive use in women. Open and closed squares and circles indicate *P* ≥ 0.05 and *P* < 0.05, respectively.

**Figure 4:**
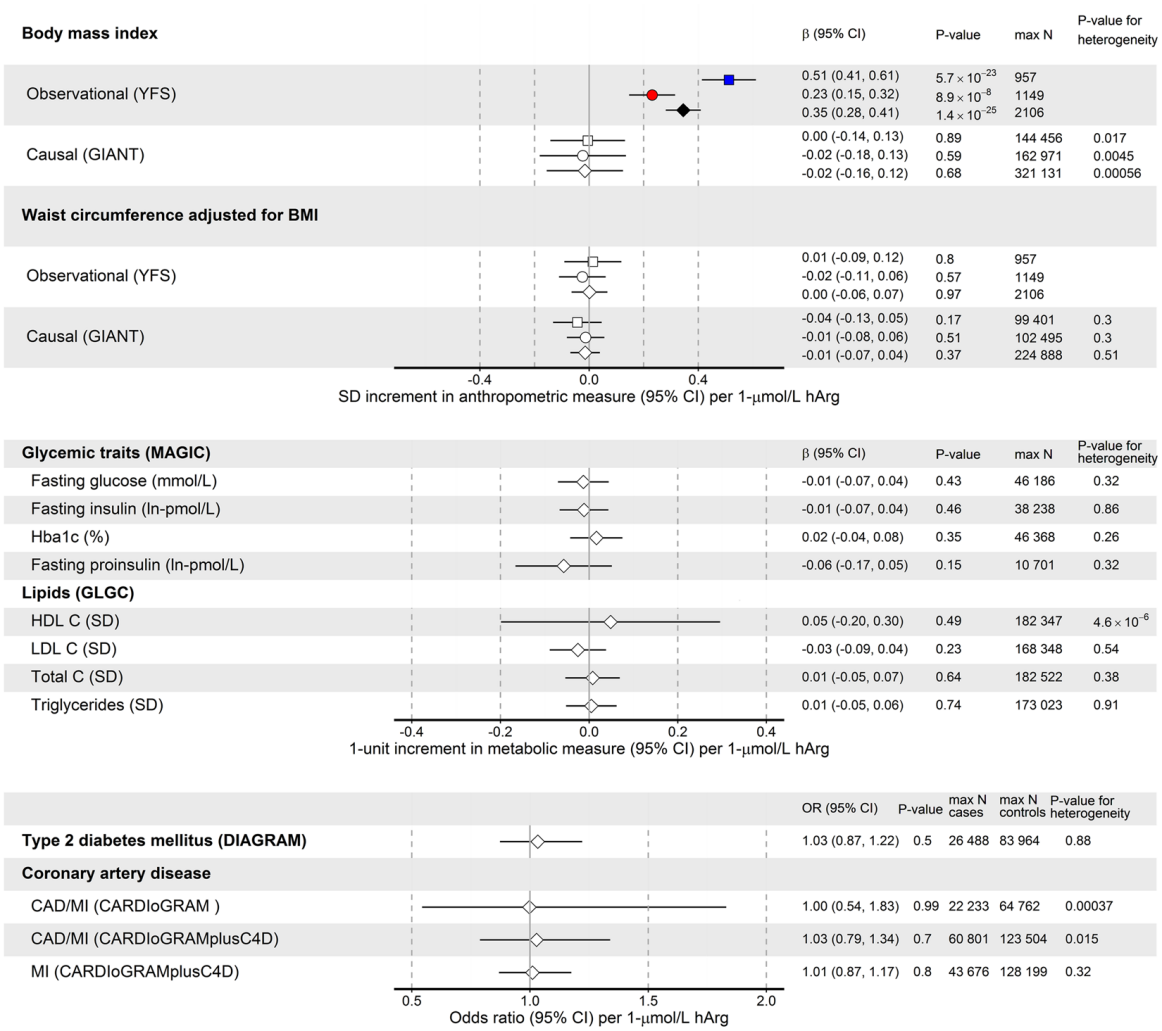
Combined causal effect estimates (β = beta, odds ratio and 95% CI confidence intervals) of hArg with cardiometabolic risk factors, type 2 diabetes mellitus (T2DM) and coronary artery disease (CAD). For each metabolite, the summary-level data across the three hArg-associated SNPs (*GATM* rs1153858, *CPS1* rs1047891 and *AGXT2* rs37369) was combined using weighted linear regression, and the heterogeneity in the causal effects from different individual variants was tested by Cochran’s Q statistic. All p-values for combined causal effects >0.05. A p-value of >0.05 from Cochran’s Q statistic indicates that there is no more heterogeneity between causal effects estimated using the variants individually than would be expected by chance.

In our 6- to 10-year follow-up (depending on the endpoints used) in unadjusted analyses, hArg was significantly and directly associated with incident obesity (BMI ≥30 kg/m^2^), abdominal obesity (high waist circumference), hyperglycaemia, high insulin, high-risk carotid intima-media thickness and distensibility in men, and with incident T2DM in women (Fig. 3). In our 10-year follow-up analysis after further adjustments with other cardiovascular risk factors, hArg served as a significant biomarker for future hyperglycaemia (OR 1.31, 95% CI 1.06–1.63) and abdominal obesity (OR 1.60, 95% 1.14–2.30) in men and predicted T2DM in women (OR 1.55, 95% CI 1.02–2.41) (Fig. 3). Furthermore, in the cross-sectional study (in 2001), for both men and women separately, hArg was strongly associated with BMI (for men P = 5.7 × 10^−23^, for women P = 8.9 × 10^−8^ and for all subjects combined P = 1.4 × 10^−26^), but not with BMI-adjusted waist circumference, in the observational analyses using the YFS data (Fig. 4).

### Estimating causal effects of hArg on obesity, lipids and glycaemic traits as well as T2DM and CAD

In this part of the study, utilizing summary-level data from different GWAS meta-analyses available in the public domain (Fig. 1), no evidence was found that serum hArg was causally associated with any of the studied obesity traits (BMI or BMI-adjusted waist circumference), glycaemic or lipid traits, or T2DM or CAD (Fig. 4).

### Estimating causal effects of hArg on 122 metabolites

**Figure 5:**
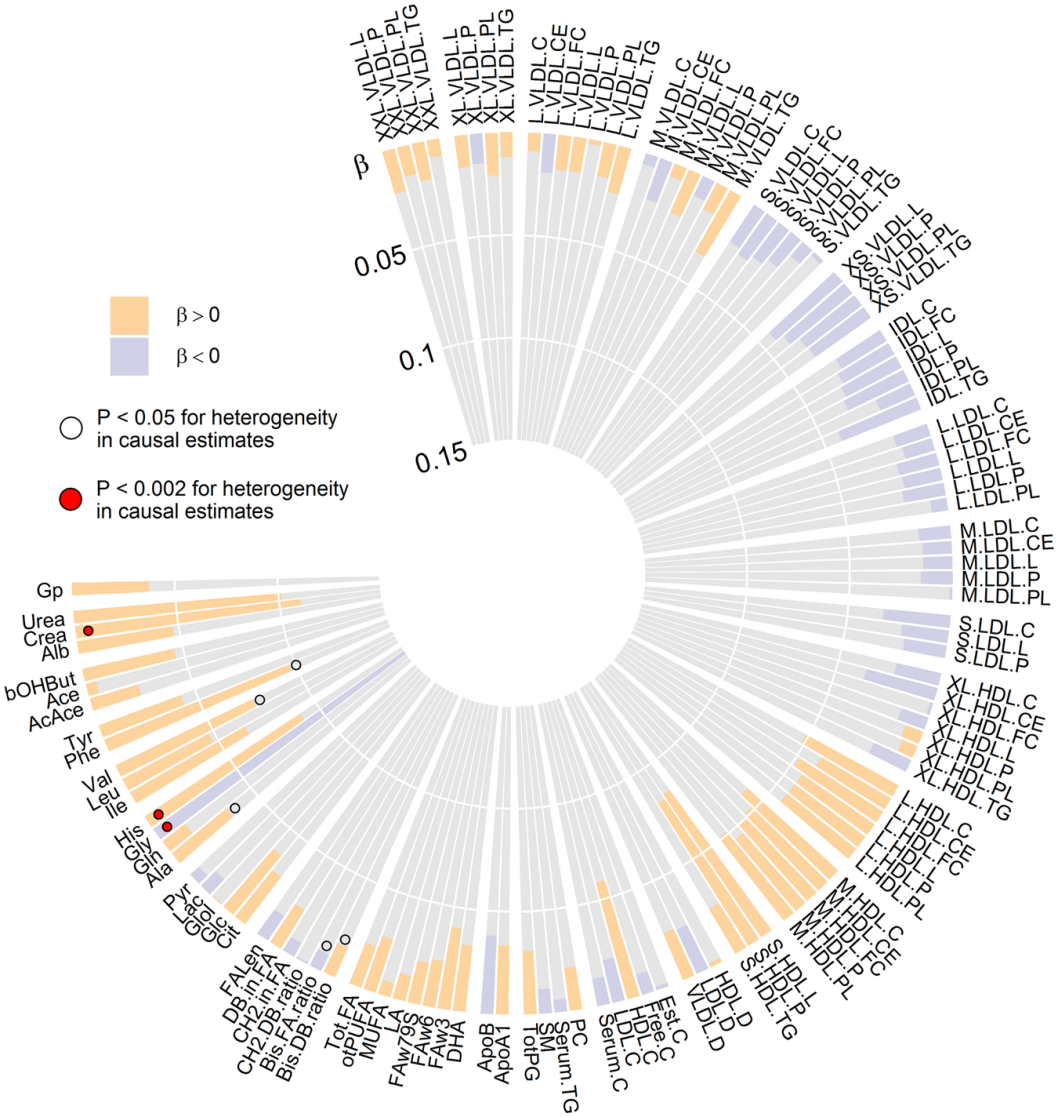
Combined causal effect estimates (β = beta; coloured bars indicate the direction and magnitude of the effect; yellow bars, β > 0, blue bars, β < 0) of hArg with 122 systemic metabolic measures using summary-level data from previous meta-analyses of genome-wide association studies. For each metabolite, the summary-level data across the three hArg associated SNPs (*GATM* rs1153858, *CPS1* rs1047891 and *AGXT2* rs37369) was combined using weighted linear regression, and the heterogeneity in the causal effects from different individual variants was tested by Cochran’s Q statistic. All p-values for combined causal effects >0.05. A p-value of >0.05 from Cochran’s Q statistic indicates that there is no more heterogeneity between causal effects estimated using the variants individually than would be expected by chance. The numbers of individuals used to estimate SNP–metabolite associations vary between 8 905 and 24 924, depending on the metabolite.

We tested the causality of serum hArg on 122 NMR-based metabolic measures, and the results of these analyses are illustrated in Fig. 5. No evidence was found of a causal relationship between hArg and any of the tested 122 metabolites (for all traits, P-value for combined causal estimates >0.05). For three metabolites (creatinine, histidine and glycine), heterogeneity in the causal estimates was detected due to strong associations with the *CPS1* variant and a lack of association or inconsistent association with the two other genetic variants (see Table S1).

## Discussion

Consistent with the positive cross-sectional association of hArg with BMI in the present and several previous studies^1, 6, 8, 13^, we now also showed that, in our 10-year follow-up analysis after adjustments with other cardiovascular risk factors, hArg served as a significant biomarker (predictor) for future hyperglycaemia and for abdominal obesity in men and incident T2DM in women. On the other hand, our MR analyses using large-scale GWAS meta-analysis summary data did not indicate causal roles for hArg in the development of high BMI or BMI-adjusted waist circumference, or any other studied clinical or metabolic trait.

In line with several previous population and patient studies^1, 6, 8, 13^, we observed a strong positive association between hArg and BMI in young adults, whereas the causal effects of hArg on BMI and waist circumference were absent. The lack of a causal role of circulating hArg in body weight is supported by an experimental study on mice and a small clinical trial showing that several-fold elevations of plasma hArg by oral hArg supplementation for several weeks did not have any effect on body weight^14, 15^. Moreover, L-arginine:glycine amidinotransferase (AGAT) -deficient mice exhibiting creatine and hArg^5^ deficiencies were completely protected from diet-induced obesity and metabolic syndrome, whereas oral creatine supplementation resulted in a complete normalisation of the body weight and composition of these animals^16^, suggesting that the effect of AGAT deficiency on BMI is hArg-independent. Therefore, it is possible that hArg is a biomarker of AGAT activity and creatine synthesis and that higher creatine levels predispose to weight gain, as suggested by the mouse model. This hypothesis is supported by our result showing that hArg has some predictive value for the development of abdominal obesity during a 10-year follow-up in young men despite careful adjustment for various baseline risk factors, including BMI. Concordantly, no change in plasma or tissue hArg concentrations was observed in a small study of patients undergoing bariatric surgery despite dramatic weight loss, suggesting that there is no causal effect of BMI on hArg levels^17^. Furthermore, the strong inverse association of baseline hArg and 10-year glycine suggests that hArg has some predictive value for creatine synthesis via AGAT (Figure S5) at least 10 years ahead of time. Taken together, hArg endogenously synthesized by AGAT might be an excellent marker of systemic creatine synthesis, explaining the positive cross-sectional association of hArg with BMI in large population and patient cohorts.

We observed a longitudinal association between baseline hArg levels and 10-year incident hyperglycaemia and a positive interaction between baseline hArg and BMI on NMR-based fasting serum glucose in young men. However, cross-sectional associations between hArg and fasting glucose were absent in both men and women, in line with the lack of causal effects on glycaemic traits in individuals without T2DM. In contrast, hArg was independently associated with fasting glucose in older adults^13^. Moreover, in an RCT on oral hArg supplementation lasting 4 weeks in 20 young volunteers, a moderate increase in plasma glucose in comparison to placebo was observed^15^. Compared to a 7-fold increase in hArg plasma levels after supplementation with 125 mg hArg once daily, the hArg-associated lead *GATM* variant used in the MR analyses showed a relatively small per allele effect of ~0.25 µmol/L (or ~14% increase from the mean value of 1.85 µmol/L). Therefore, the possible effect of hArg on blood glucose is likely seen at much higher concentrations of hArg than what is normally present in the blood, in line with the tightly regulated glucose homeostasis in humans without overt T2DM. A 16-week oral hArg supplementation resulted in a 6-fold increase in plasma hArg, as well as increased insulin secretion and reduced blood glucose in mice on a high-fat diet but not in mice on a normal diet^14^. This contrasts with a moderate increase in blood glucose in the RCT, probably due to the differences in glucose metabolism between mice and humans.

In concordance with several previous studies^8, 13^, we observed an independent negative association between daily smoking and circulating hArg levels, while the molecular underpinnings of this association remain elusive. In contrast to some studies on older adults from general and patient populations^6, 13^, we observed no associations between baseline hArg and incident arterial hypertension during a 10-year follow-up, or baseline diastolic or systolic blood pressure in young adults. We were unable to study the causality of lifelong exposure to hArg on blood pressure due to the lack of publicly available complete summary statistics on the hArg-associated SNPs, and further MR studies are thus warranted to address whether there is any causal effect of circulating hArg on blood pressure in humans. However, the RCT on hArg supplementation in young volunteers did not detect any differences in diastolic or systolic blood pressure between hArg and placebo after a 4-week supplementation^15^.

Baseline hArg was positively associated with incident T2DM especially in women, although the association was attenuated after adjusting for cardiometabolic risk factors and the use of oral contraceptives. A causal effect of hArg on T2DM was absent, suggesting that lifelong exposure to higher circulating hArg levels does not increase the risk of developing T2DM. In some population and patient cohorts, observational cross-sectional associations between hArg and prevalent T2DM were absent^6, 7, 18^, whereas in some studies, T2DM was associated with lower hArg levels compared to non-diabetic controls^8^, and, in patients with T2DM undergoing maintenance haemodialysis, low hArg levels correlated with longer duration of T2DM^1^.

No independent associations were observed between hArg and 6-year incident high-risk carotid intima-media thickness or carotid distensibility. Neither was the baseline hArg associated with the presence of coronary artery calcification assessed seven years after the hArg quantification. These results are consistent with the lack of a cross-sectional association of hArg with coronary calcium in the population-based Dallas Heart Study^8^. In addition, there were no associations between genetically determined hArg and CAD or myocardial infarction, suggesting that serum hArg does not have a causal effect on the development of coronary atherosclerosis or its clinical manifestations in humans, at least at concentrations normally observed in the circulation. This is in line with the lack of a cross-sectional association between hArg and prevalent CAD in over 3000 patients referred for coronary angiography^18^ as well as with the absence of associations between baseline hArg and future cardiovascular events and myocardial infarction in haemodialysis patients^2^. The experimental evidence on the role of hArg in atherosclerosis is not conclusive, as one study showed that hArg augments the osteo-/chondrogenic transformation of human aortic vascular smooth muscle cells and aortic calcification^9^, whereas another study reported a beneficial role of hArg in balloon-injured rat carotids trough the inhibition of neointimal formation^19^.

There are no earlier systematic studies investigating the causal role of hArg in the regulation of serum metabolite profiles. In our analysis, we found no evidence for causal relationships between circulating hArg and 122 systemic metabolite measures. There are several potential explanations for this. Firstly, it is possible that hArg is an innocent AGAT metabolite and, at best, a biomarker with no direct causal effects on systemic metabolic pathways or diseases. This view is supported by experiments in rats and pigs, showing that >95% of orally administered hArg was recovered in urine and not metabolized in tissues^20^. Moreover, the RCT on oral hArg supplementation in 20 young volunteers did not detect any systemic effects, with the exception of a moderate increase in blood glucose, despite a 7-fold increase in plasma hArg compared to placebo^15^. Secondly, hArg may have specific effects on metabolic pathways not covered by the metabolomics platform used or may play a more important role in certain pathologies rather than in healthy individuals.

The use of oral oestrogen contraceptives in young women was associated with a marked increase in hArg levels. A previous study of pregnant YFS participants reported that hArg levels were increased during the second and third trimesters of normal pregnancy when compared to non-pregnant women and that hArg was positively correlated with gestational age^21^. The gradual increase in hArg levels during normal pregnancy is likely due to a parallel increase in maternal oestrogen levels. This reasoning is supported by experiments showing that the expression of the *AGAT* gene is modulated by oestrogen in chick liver^22^ and consistent with a marked increase in the renal *AGAT* mRNA and protein expression of pregnant spiny mice at term when compared to non-pregnant animals^23^. The fact that the hArg synthesis by AGAT is modulated by sex hormones might aid to explain the observed sex differences in the associations between hArg and incident cardiometabolic risk factors and T2DM. This hypothesis warrants further examination.

Our study has limitations that are general to all MR studies, and these should be considered when interpreting the results. The three MR assumptions in the context of the present study are as follows: 1) the genetic variants should be associated with the circulating hArg levels, 2) they should affect the outcome only through the circulating hArg levels (exclusion restriction assumption), and 3) they should be independent of hArg-outcome confounders (independence assumption). Regarding the first assumption, we acknowledge that the sample size used to quantify the strength of the SNP–hArg associations is limited (n = 5143). On the other hand, the lead *GATM* variant is a strong instrument for MR analyses, explaining ~5.3% of the variability in serum hArg levels in YFS, and all three variants included in the MR analyses showed genome-wide significant (P < 5 × 10^−8^, F-statistic >120, Table S2) associations with serum hArg levels in the original GWAS^24^. In addition, the statistical power in two-sample MR analyses is mainly determined by the number of individuals used to assess SNP–outcome associations^25^, for which we used publicly available summary statistics from large consortiums involving tens to hundreds of thousands of individuals. An adequate statistical power to detect causal effects is evident by the inspection of the 95% confidence intervals for, e.g., BMI-adjusted waist circumference in Fig. 4 showing that the confidence intervals for the combined causal estimates are comparable to those from the observational association analyses when there is no heterogeneity between the causal estimates. Moreover, some degree of sample overlap between individuals used to estimate SNP–hArg and SNP–outcome associations is present for some of the outcomes studied; however, the possible effect of this sample overlap on the causal estimates is likely to be small, because the individuals used in the hArg GWAS constitute a very small proportion of the total number of individuals used in the large meta-analyses of GWASs on outcomes. The risk that the independence assumption is violated due to a population stratification in GWASs is minimized by adjustments for principal components. Another potential concern in our MR analyses, as in all MR studies, is a horizontal pleiotropy (i.e. the genetic variants affect the outcome through pathways not mediated by hArg levels). We could avoid too precise causal estimates in the cases in which some of the genetic variants showed a pleiotropic effect on outcome by using weighted linear regression, and formally tested the presence of heterogeneity between the causal estimates using Cochran’s Q statistic. Moreover, the three genome-wide significant hArg-related SNPs used to estimate causal effects have biologically plausible and functional roles in the endogenous hArg synthesis and/or catabolism as illustrated in Figures S6 and S7. Finally, despite single-variant pleiotropy in some SNP–outcome associations, the other two of the three independent variants showed not even a weak association with the outcome (Tables S1 and S3–S7). As discussed before^26^, although both negative and positive results are potentially subject to a violation of the assumptions required for MR, in the case of negative results, the biases would have to balance out perfectly to result in an effect estimate of zero when there is in fact a true effect, making negative findings from MR more reliable than positive ones.

In the observational analyses, the 10-year follow-up was not long enough to investigate very long term predictive value of hArg on studied cardiometabolic outcomes. However, we observed a significant association between baseline hArg and new onset T2DM in young women indicating that the follow-up period was sufficiently long to detect such associations despite limited number of new-onset cases of T2DM. Furthermore, the follow-up period of 6 years used to study the association between hArg and high-risk carotid atherosclerosis is relatively short. However, traditional cardiometabolic risk factors were associated with these outcomes suggesting that the follow-up time was long enough to detect such association also with hArg.

In conclusion, our results show that circulating hArg is not causally associated with various cardiometabolic risk factors, the systemic metabolic profile, T2DM or CAD. Our results do not provide any consistent evidence that interventions aimed at modifying hArg levels will improve the metabolic profiles or risk of T2DM or CAD. Whether hArg could be used as a biomarker to predict future abdominal obesity in young men remains to be investigated in future studies.

## Methods

### Study population and data sources

YFS is a Finnish longitudinal population study on the evolution of cardiovascular risk factors from childhood to adulthood^27^. The study began in 1980, when 3,596 children and adolescents aged 3–18 years were randomly selected from five university hospital catchment areas in Finland. In 2001, 2,288 participants aged 24–39 years attended the 21-year follow-up. We excluded 33 pregnant women, as their hArg levels have been shown to be elevated during pregnancy in the YFS^21^, as well as 11 subjects who had type 1 diabetes at baseline and one participant who had an exceptionally high hArg serum concentration (20.2 µmol/L) from all analyses. Therefore, 2,106 participants contributed to the cross-sectional association analysis of hArg and cardiometabolic risk factors at baseline (in 2001). We considered the participants of the longitudinal analyses who attended the 2001 follow-up and at least one later follow-up in 2007 or 2011. Of these participants, we included those for whom hArg and covariate data at the 2001 follow-up (used as baseline for the present study) and cardiometabolic risk factor data at the 2007 and/or 2011 follow-up were available and who were not pregnant in 2001 (n = 1801). The YFS was approved by the 1st ethical committee of the Hospital District of Southwest Finland (on September 21st, 2010) and by local ethical committees. The methods were carried out in accordance with the relevant guidelines and regulations. The participants gave written informed consent.

### Genetic instrument selection for Mendelian randomization (MR) studies

For MR analyses, we used three single-nucleotide polymorphisms (SNPs) – *GATM* (glycine amidinotransferase) rs1153858, *CPS1* (carbamoyl-phosphate synthase 1) rs1047891 (formerly rs7422339), and *AGXT2* (alanine-glyoxylate aminotransferase 2) rs37369 – that were all associated with serum hArg levels on a genome-wide significance level (P < 5 × 10^−8^) in a recent genome-wide association study (GWAS) of 5,143 individuals^24^. The *GATM* rs1153858 variant alone, also associated with *GATM* mRNA expression in a tissue-specific manner (see Figure S6), explained ~5.3% of the variability in serum hArg levels in the YFS^24^. The summary statistics for the SNP-outcome associations were extracted from the summary-level data available in the public domain from large GWASs (see number of subjects in Fig. 1) investigating the associations of gene variants with conventional cardiometabolic risk factors, including BMI^28, 29^, waist circumference^30^, fasting glucose^31^, fasting insulin^31^, HbA_1_c^32^, proinsulin^33^, total cholesterol^34^, high-density lipoprotein (HDL) cholesterol^34^, triglycerides^34^, low-density lipoprotein (LDL) cholesterol^34^ as well as CAD^35, 36^ and T2DM^37^. The summary-level data for 122 NMR-method-based serum metabolites from the MAGNETIC NMR GWAS consortium^38^ were downloaded from http://computationalmedicine.fi/data. All associations extracted from the summary-level data on hArg-related variants and outcomes are presented in Tables S1–S7.

### Serum hArg measurements and other biochemical assays

HArg was measured in serum stored at −80 °C by means of reversed-phase high-performance liquid chromatography at the Clinical Institute of Medical and Chemical Laboratory Diagnostics, Medical University of Graz^39^. Details of the biochemical measurements and clinical data are presented in Supplementary Methods.

### Metabolic profiling

High-throughput NMR spectroscopy was used for the absolute quantification of serum metabolites. The metabolite set (includes 228 quantified metabolites) covers multiple metabolic pathways, including lipoprotein lipids and subclasses, fatty acids and fatty acid compositions, as well as amino acids and glycolysis precursors. All molecular measures are quantified in a single experimental setup, constituting both established and novel metabolic risk factors. This NMR-based metabolite profiling has previously been used in various epidemiological^40, 41^ and genetic studies^38^ and has been reviewed recently^42^. Details of the experimentation have been described elsewhere^42, 43^.

### Incident outcome definitions for the studied traits

In the YFS, the metabolic syndrome components were defined according to harmonized criteria^11^: waist circumference ≥102 cm in males and ≥88 cm in females (1), hypertriglyceridemia >1.7 mmol/l (2), HDL cholesterol <1.0 mmol/l in males and <1.3 mmol/l in females (3), blood pressure ≥130/85 mmHg or treatment for blood pressure (4), and fasting plasma glucose ≥5.6 mmol/l or previously diagnosed T2DM. High insulin was defined as a 10-year fasting insulin in the >90th percentile using sex-specific values corresponding with a fasting insulin of ≥18.8 IU/L for men and ≥15.2 IU/L for women. Obesity was defined as BMI ≥30 kg/m^2^. In 2008, cardiac computed tomography was performed to measure coronary artery calcification (CAC) for a subsample of 589 participants then aged 40–46 years^44^. The absence of CAC was defined as an Agatston score of 0, and individuals with an Agatston score of 1 or greater were classified as having CAC. In 2001 and 2007, carotid artery intima-media thickness (IMT) and distensibility were measured as described in detail previously^45, 46^. As previously^47^, high-risk carotid IMT and distensibility were defined as falling within the age- and sex-specific ≥90 percentile and ≤10 percentile, respectively. Participants were classified as having T2DM if they had fasting a plasma glucose level of 7.0 mmol/l or greater, reported the use of oral glucose-lowering medication or insulin but had not reported having type 1 diabetes mellitus, or reported a diagnosis of T2DM by a physician. Participants were also classified as having T2DM if they had HbA1c ≥6.5% (48 mmol/mol).

### Statistical Methods

All statistical analyses were conducted with R version >3.1.2 (https://www.r-project.org/).

### Clinical determinants of hArg levels in YFS

We used linear regression analysis to explore the associations of hArg with common cardiovascular risk factors at baseline (age, sex, LDL cholesterol, HDL cholesterol, triglycerides, systolic and diastolic blood pressure, CRP, glucose, insulin, BMI, waist circumference, smoking and family history of CAD) as well as serum sex hormone-binding globulin (SHBG) and hormonal oral contraceptive use in women. Stepwise model selection by Akaike’s information criterion (AIC) was performed to determine the most important determinants of hArg levels using the stepAIC R function.

### Cross-sectional association analyses of hArg and metabolites in YFS

To facilitate the log-transformation, zero values in NMR-based metabolic measures were replaced by half of the minimum positive value found for each metabolite, assuming this to be the best estimation of detection limit. Prior to statistical analyses, NMR-based metabolic measures were log-transformed and scaled to standard deviations (SD) to facilitate comparisons across metabolites. Association magnitudes are reported in SD units of metabolite concentration per 1-SD increment in log-transformed hArg. For sex-stratified cross-sectional analysis, sex-specific scaling was applied for NMR metabolites. Due to the correlated nature of the systemic metabolite measures, over 95% of the variation in the metabolic data was explained by 25 principal components, and a P-value of 0.05/25 = 0.002 was thus required after multiple testing correction^40^.

Cross-sectional and longitudinal associations of baseline hArg with circulating metabolites at baseline and at the follow-ups were analysed using linear regression models, stratified by sex, with each metabolic measure as the outcome and hArg as the explanatory variable. The regression models were adjusted for age, BMI, daily smoking, serum SHBG and oral contraceptive use (in women). For each metabolic measure, the sex-related differences in observational estimates were tested by using the Z-statistic. Interactions between BMI and each metabolite measure in relation to hArg levels were tested in men and women separately by adding the interaction term to the linear regression models.

### Longitudinal association analyses of hArg with cardiometabolic risk factors, preclinical atherosclerosis and T2DM in YFS

The prospective associations of hArg with 6- or 10-year incident Mets components, high insulin (see incident outcome definitions above), and incident obesity (BMI >30 kg/m^2^) were assessed using logistic regression models with scaled log-transformed baseline hArg as an explanatory variable. Incident T2DM during the 10-year follow-up was used as an outcome variable to assess the prospective association of hArg with T2DM. The follow-up period for carotid artery ultrasound parameters was six years. Coronary calcification was assessed once seven years after the baseline in 2008. Those with the condition at baseline were excluded from the analysis. We fitted unadjusted models (Model 1) and models that were adjusted for all cardiometabolic risk factors shown in Table 1 (Model 2) and, further, for serum SHBG and oral contraceptive use (in women, Model 3).

### Instrumental variable analyses using publicly available summary-level data on hArg, metabolites, cardiometabolic traits, T2DM and CAD

The strengths of the genetic instruments used for hArg were assessed based on the F-statistic derived from the summary statistics of the hArg GWAS (Table S2). Despite the relatively small sample size (n = 5143) in the meta-analysis of GWASs on hArg^24^, all F-statistics for the instruments were well over 10 (between 120 and 370) (Table S2), indicating that all three instruments separately are sufficiently strong to be used in MR studies. We then used these instruments in the Mendelian randomization (MR) analyses to quantify the strengths of the causal associations of hArg with NMR metabolites, cardiometabolic traits, T2DM and CAD. As previously^48^, to evaluate combined causal estimates with 95% confidence intervals (95% CI) from summary statistics of the three independent SNPs, we performed a weighted linear regression of the genetic associations with each outcome variable on the genetic associations with hArg using first-order weights described in more detail with an R code implementation elsewhere^49^. This multiplicative random-effects model was used for combining causal estimates to obtain the same point estimate as from a fixed-effects model while avoiding too precise estimates in the case of heterogeneity in the causal effects obtained from different genetic variants. The combined MR estimates are in units of 1-unit increment of a continuous outcome (or odds ratio of disease risk) per 1-µmol/L increment in hArg. We performed heterogeneity tests based on Cochran’s Q statistic to detect potential dissimilarity between the causal effects across the hArg SNPs (instrumental variables).

## Electronic supplementary material

Supplementary Information
