# Supplementary material for: The biomarker and causal roles of homoarginine in the development of cardiometabolic diseases: an observational and Mendelian randomization analysis
Source: Sci Rep. 2017 Apr 25;7:1130. doi: 10.1038/s41598-017-01274-6 (PMC5430630; doi:10.1038/s41598-017-01274-6)

Supplementary Online Contents

# The biomarker and causal roles of homoarginine in the development of cardiometabolic diseases: an observational and Mendelian randomization analysis

Ilkka Seppälä, Niku Oksala, Antti Jula, Antti J. Kangas, Pasi Soininen, Nina Hutri-Kähönen, Winfried März, Andreas Meinitzer, Markus Juonala, Mika Kähönen, Olli T. Raitakari, Terho Lehtimäki

*Supplementary Methods*

**The cardiovascular risk in the Young Finns Study (YFS)**

*Supplementary Tables*

**Table S1. Association of hArg related genetic variants with metabolites in the MAGNETIC NMR GWAS.**

**Table S2. Association of hArg related genetic variants with serum hArg in a GWAS (n=5143).**

**Table S3. Association of hArg related genetic variants with BMI in GIANT.**

**Table S4. Association of hArg related genetic variants with waist circumference adjusted for BMI in GIANT.**

**Table S5. Association of hArg related genetic variants with glycemic traits in MAGIC.**

**Table S6. Association of hArg related genetic variants with blood lipids in GLGC.**

**Table S7. Association of hArg related genetic variants with T2DM and CAD in DIAGRAM and CARDIoGRAMplusC4D.**

*Supplementary Figures*

**Figure S1. Serum concentrations of homoarginine (hArg) (µmol/L) by the use of different hormonal contraceptive methods.**

**Figure S2. Cross-sectional and longitudinal associations of baseline hArg with all 228 metabolites for both sexes separately.**

**Figure S3. Cross-sectional and longitudinal associations of baseline hArg with all 228 metabolites for both sexes combined.**

**Figure S4. Cross-sectional association of hArg with 73 metabolites adjusted for age, BMI and daily smoking.**

**Figure S5. Tissue-specific *GATM* mRNA expression and *GATM* rs1153858.**

**Figure S6. A hypothetical model of hArg metabolism in humans.**

## Supplementary Methods

### The cardiovascular risk in the Young Finns Study (YFS)

*Biochemical measurements*

For hArg quantification, intraday coefficients of variation (CVs) at different concentrations (mean levels) were 4.7% (1.21 µmol/L) and 2.2% (3.53 µmol/L), and between-day CVs were 7.9% (1.25 µmol/L) and 6.8% (3.66 µmol/L), respectively [1].

Venous blood samples were drawn after a 12 h fast. Serum triglycerides, total cholesterol, high density lipoprotein (HDL)-cholesterol, were measured as described previously [2]. Low density lipoprotein (LDL)-cholesterol was calculated using the Friedewald formula for participants with triglycerides <4 mmol/l. Glucose concentrations were analyzed enzymatically with a clinical chemistry analyzer (Olympus, AU400), and serum insulin concentrations were measured by microparticle enzyme immunoassay kit (Abbott Laboratories, Diagnostic Division, Dainabot). Serum C-reactive protein (CRP) was analyzed by an automated analyzer (Olympus AU400) with a latex turbidimetric immunoassay kit (CRP-UL assay, Wako Chemicals, Neuss, Germany). The detection limit reported by the manufacturer for the assay was 0.06 mg/l. Sex hormone-binding globulin (SHBG) was measured by Spectria SHBG IRMA.

*Clinical measurements and questionnaires*

Height, weight and waist circumference were measured. BMI was calculated using the formula: weight [kg]/(height [m])$^2$. Blood pressure was measured using a random zero sphygmomanometer with the average of three measurements used in the analyses. Participants were also asked to complete questionnaires that included questions on smoking habits and family history of premature CAD.

## *Supplementary Tables*

**Table S1. Association of hArg related genetic variants with metabolites in the MAGNETIC NMR GWAS.**

| | Glycine | | Histidine | | Creatinine | | Phenylalanine | |
|---|---|---|---|---|---|---|---|---|
| SNP (effect allele)[a] | Beta[b] (SE) | N P value | Beta (SE) | N P value | Beta (SE) | N P value | Beta (SE) | N P value |
| rs1047891 (C) | -0.49 (0.011) | 18 730 0 | -0.060 (0.011) | 19 241 $1.1 \times 10^{-7}$ | -0.048 (0.010) | 24 805 $3.3 \times 10^{-6}$ | 0.038 (0.010) | 22 657 $3.1 \times 10^{-4}$ |
| rs37369 (T) | -0.0018 (0.019) | 16 507 0.93 | -0.027 (0.017) | 19 244 0.11 | -0.0034 (0.016) | 22 583 0.83 | -0.0051 (0.017) | 20 436 0.76 |
| rs1153858 (T) | -0.0053 (0.011) | 18 733 0.64 | 0.012 (0.011) | 19 243 0.27 | 0.080 (0.010) | 24 809 $8.3 \times 10^{-15}$ | 0.020 (0.010) | 22 662 0.053 |

Shown are the metabolites that are associated with at least one of the hArg related SNPs at P<0.001.
a. Effect allele is the hArg increasing allele.
b. Betas are in the units of 1-SD increment in metabolic measure per effect allele.
Betas and standard errors obtained from MAGNETIC NMR GWAS [5] and downloaded from http://computationalmedicine.fi/data.

**Table S2. Association of hArg related genetic variants with serum hArg in a GWAS (n=5143).**

| SNP | Gene | Chr | Effect allele[a] | Effect allele frequency (%) | Increase in hArg (µmol/L) per effect allele[d] | Standard error[d] | P value[d] | R statistic (%)[e] | F statistic[f] |
|---|---|---|---|---|---|---|---|---|---|
| rs1047891[b] | CPS1 | 2 | C | 69.8 | 0.16 | 0.020 | $6.5 \times 10^{-17}$ | 4.4 | 240 |
| rs37369 | AGXT2 | 5 | T | 8.6 | 0.22 | 0.030 | $7.9 \times 10^{-14}$ | 2.2 | 120 |
| rs1153858 | GATM | 15 | T | 27.6 | 0.26 | 0.018 | $4.1 \times 10^{-48}$ | 6.8 | 370 |

a. Effect allele is the hArg increasing allele.
b. Formerly rs7422339.
c. Effect allele frequency in individuals of European decent of the 1000 Genomes project.
d. Combined effect estimates and standard errors in the units of 1-µmol/L increment in hArg per effect allele were calculated by fixed-effects meta-analysis from the study-specific summary statistics taken from Kleber et al. [3]
e. The proportion of variance in hArg explained by the SNP (the $R^2$ statistic) is approximately equal to $2\beta \times MAF \times (1 - MAF)$, where SNP-hArg β is given in standard deviation units and MAF is minor allele frequency. [4] To convert the βs into standard deviation units, we assume that 1-SD equals approximately 0.65 µmol/L hArg as we observe in YFS (**Table 1**):

$$\beta\ (SD/effect\ allele) = 0.65 \times \beta\ (\mu mol/L/effect\ allele).$$

f. The F statistic can then be calculated from the $R^2$ statistic as $F = \frac{N-K-1}{K}\frac{R^2}{1-R^2}$, where N is the sample size and K is the number of genetic variants (here K=1). [4]

**Table S3. Association of hArg related genetic variants with BMI in GIANT.**

| | Men | | Women | | Combined | |
|---|---|---|---|---|---|---|
| SNP (effect allele)[a] | Beta[b] (SE) | N P value | Beta (SE) | N P value | Beta (SE) | N P value |
| **Any age** | | | | | | |
| rs1047891 (C) | -0.011 (0.0047) | 144 456 0.020 | -0.0176 (0.0046) | 160 979 $1.5 \times 10^{-4}$ | -0.014 (0.0034) | 317 601 $4.6 \times 10^{-5}$ |
| rs37369 (T) | -0.0039 (0.0073) | 143 800 0.59 | 0.0013 (0.0072) | 162 971 0.86 | -0.0027 (0.0051) | 321 131 0.60 |
| rs1153858 (T) | 0.0096 (0.0057) | 104 566 0.092 | 0.0026 (0.0053) | 129 057 0.62 | 0.0060 (0.0041) | 233 074 0.14 |

a. Effect allele is the hArg increasing allele.
b. Betas are in the units of 1-SD increment per effect allele. Betas and standard errors obtained from BMI GWASs of the GIANT consortium [6,7] and downloaded from http://portals.broadinstitute.org/collaboration/giant/index.php/GIANT_consortium_data_files.

**Table S4. Association of hArg related genetic variants with waist circumference adjusted for BMI in GIANT.**

| | Men | | Women | | Combined | |
|---|---|---|---|---|---|---|
| SNP (effect allele)[a] | Beta[b] (SE) | N P value | Beta (SE) | N P value | Beta (SE) | N P value |
| rs1047891 (C) | -0.0012 (0.0059) | 83 402 0.84 | 0.0044 (0.0053) | 102 495 0.41 | 0.0008 (0.004) | 188 157 0.84 |
| rs37369 (T) | -0.0055 (0.0083) | 99 401 0.51 | 0.0038 (0.0074) | 123 454 0.61 | -0.0016 (0.0056) | 224 888 0.77 |
| rs1153858 (T) | -0.019 (0.0071) | 61 417 0.0071 | -0.0012 (0.0059) | 89 868 0.84 | -0.0075 (0.0047) | 151 092 0.11 |

a. Effect allele is the hArg increasing allele.
b. Betas are in the units of 1-SD increment per effect allele.
Betas and standard errors obtained from a GWAS of the GIANT consortium [8] and downloaded from http://portals.broadinstitute.org/collaboration/giant/index.php/GIANT_consortium_data_files.

**Table S5. Association of hArg related genetic variants with glycaemic traits in MAGIC.**

| | Fasting glucose (mmol/L) max N = 46 186 | | Fasting insulin (ln-pmol/L) max N = 38 238 | | Hba1c (%) max N = 46 368 | | Fasting proinsulin (ln-pmol/L) max N = 10 701 | |
|---|---|---|---|---|---|---|---|---|
| SNP (effect allele)[a] | Beta[b] (SE) | P value | Beta (SE) | P value | Beta (SE) | P value | Beta (SE) | P value |
| rs1047891 (C) | 0.0051 (0.0044) | 0.25 | -0.0039 (0.0046) | 0.40 | 0.0081 (0.0043) | 0.059 | -0.0004 (0.0077) | 0.96 |
| rs37369 (T) | 0.0060 (0.0067) | 0.37 | -0.0004 (0.0069) | 0.96 | 0.0068 (0.0062) | 0.27 | -0.025 (0.013) | 0.054 |
| rs1153858 (T) | 0.0046 (0.0041) | 0.25 | -0.0026 (0.0042) | 0.53 | 0.0006 (0.0038) | 0.88 | -0.017 (0.0079) | 0.036 |

a. Effect allele is the hArg increasing allele.
b. Betas are in the units of 1-unit increment in glycaemic trait per effect allele.
Betas and standard errors obtained from the GWASs of the MAGIC consortium [9-11] and downloaded from https://www.magicinvestigators.org/downloads/.

**Table S6. Association of hArg related genetic variants with blood lipids in GLGC.**

| | Total cholestrol | | LDL cholesterol | | HDL cholesterol | | Triglycerides | |
|---|---|---|---|---|---|---|---|---|
| SNP (effect allele)[a] | Beta[b] (SE) | N P value | Beta (SE) | N P value | Beta (SE) | N P value | Beta (SE) | N P value |
| rs1047891 (C) | 0.0046 (0.0040) | 182217 0.18 | -0.0079 (0.0042) | 168110 0.14 | 0.0269 (0.0039) | 182043 $8.7 \times 10^{-10}$ | 0.0000 (0.0038) | 172729 0.86 |
| rs37369 (T) | 0.0042 (0.0063) | 182522 0.43 | -0.0016 (0.0066) | 168348 0.98 | 0.0017 (0.0061) | 182347 0.63 | 0.0033 (0.0059) | 173023 0.61 |
| rs1153858 (T) | -0.0041 (0.0058) | 91464 0.52 | -0.0046 (0.0059) | 86847 0.41 | -0.0043 (0.0054) | 91229 0.46 | 0.0008 (0.0053) | 87882 0.70 |

a. Effect allele is the hArg increasing allele.
b. Betas are in 1-SD units per effect allele.
Betas and standard errors obtained from the GLGC GWAS [12] and downloaded from http://csg.sph.umich.edu//abecasis/public/lipids2013/.

**Table S7. Association of hArg related genetic variants with T2DM and CAD in DIAGRAM and CARDIoGRAMplusC4D.**

| | **Type 2 diabetes mellitus DIAGRAM** | | **Coronary artery disease and/or myocardial infarction 22 233 cases/ 64 762 controls CARDIoGRAM** | | **Coronary artery disease and/or myocardial infarction 60 801 cases/ 123 504 controls CARDIoGRAMplusC4D** | | **Myocardial infarction 43 676 cases/ 128 199 controls CARDIoGRAMplusC4D** | |
|---|---|---|---|---|---|---|---|---|
| SNP (effect allele)[a] | OR[b] (95% CI) | N P value | log odds (SE) | Cases Controls P value | log odds (SE) | P value | log odds (SE) | P value |
| rs1047891 (C) | 1.01 (0.97-1.04) | 64 422 0.74 | 0.047 (0.019) | 13 386 53 366 0.013 | 0.025 (0.011) | 0.018 | 0.011 (0.012) | 0.34 |
| rs37369 (T) | 1.00 (0.97-1.04) | 106 228 0.86 | 0.053 (0.025) | 20 575 58 574 0.025 | 0.022 (0.014) | 0.11 | 0.014 (0.015) | 0.34 |
| rs1153858 (T) | 1.01 (0.98-1.03) | 110 198 0.62 | -0.039 (0.016) | 21 654 61 954 0.013 | -0.012 (0.010) | 0.23 | -0.0087 (0.011) | 0.44 |

a. Effect allele is the hArg increasing allele.
b. Betas are in the units of OR or log odds per effect allele.
Data on coronary artery disease / myocardial infarction have been contributed by CARDIoGRAMplusC4D investigators [13,14] and have been downloaded from www.CARDIOGRAMPLUSC4D.ORG.
Data on T2DM have been contributed by DIAGRAM investigators [15] and have been downloaded from http://diagram-consortium.org/downloads.html.

## Supplementary References

1. Meinitzer A, Puchinger M, Winklhofer-Roob BM, Rock E, Ribalta J, Roob JM, et al. Reference values for plasma concentrations of asymmetrical dimethylarginine (ADMA) and other arginine metabolites in men after validation of a chromatographic method. Clin Chim Acta 2007;384:141-148.

2. Raiko JRH, Viikari JSA, Ilmanen A, Hutri-Kähönen N, Taittonen L, Jokinen E, et al. Follow-ups of the Cardiovascular Risk in Young Finns Study in 2001 and 2007: levels and 6-year changes in risk factors. J Intern Med 2010;267:370-384.

3. Kleber ME, Seppälä I, Pilz S, Hoffmann MM, Tomaschitz A, Oksala N, et al. Genome-wide association study identifies 3 genomic loci significantly associated with serum levels of homoarginine: the AtheroRemo Consortium. Circ Cardiovasc Genet 2013;6:505-513.

4. Burgess S, Dudbridge F, Thompson SG. Combining information on multiple instrumental variables in Mendelian randomization: comparison of allele score and summarized data methods. Stat Med 2016;35:1880-1906.

5. Kettunen J, Demirkan A, Würtz P, Draisma HHM, Haller T, Rawal R, et al. Genome-wide study for circulating metabolites identifies 62 loci and reveals novel systemic effects of LPA. Nat Commun 2016;7:11122.

6. Winkler TW, Justice AE, Graff M, Barata L, Feitosa MF, Chu S, et al. The Influence of Age and Sex on Genetic Associations with Adult Body Size and Shape: A Large-Scale Genome-Wide Interaction Study. PLoS Genet 2015;11:e1005378.

7. Locke AE, Kahali B, Berndt SI, Justice AE, Pers TH, Day FR, et al. Genetic studies of body mass index yield new insights for obesity biology. Nature 2015;518:197-206.

8. Shungin D, Winkler TW, Croteau-Chonka DC, Ferreira T, Locke AE, Mägi R, et al. New genetic loci link adipose and insulin biology to body fat distribution. Nature 2015;518:187-196.

9. Dupuis J, Langenberg C, Prokopenko I, Saxena R, Soranzo N, Jackson AU, et al. New genetic loci implicated in fasting glucose homeostasis and their impact on type 2 diabetes risk. Nat Genet 2010;42:105-116.

10. Soranzo N, Sanna S, Wheeler E, Gieger C, Radke D, Dupuis J, et al. Common variants at 10 genomic loci influence hemoglobin $A_1$(C) levels via glycemic and nonglycemic pathways. Diabetes 2010;59:3229-3239.

11. Strawbridge RJ, Dupuis J, Prokopenko I, Barker A, Ahlqvist E, Rybin D, et al. Genome-wide association identifies nine common variants associated with fasting proinsulin levels and provides new insights into the pathophysiology of type 2 diabetes. Diabetes 2011;60:2624-2634.

12. Willer CJ, Schmidt EM, Sengupta S, Peloso GM, Gustafsson S, Kanoni S, et al. Discovery and refinement of loci associated with lipid levels. Nat Genet 2013;45:1274-1283.

13. Schunkert H, König IR, Kathiresan S, Reilly MP, Assimes TL, Holm H, et al. Large-scale association analysis identifies 13 new susceptibility loci for coronary artery disease. Nat Genet 2011;43:333-338.

14. Nikpay M, Goel A, Won H, Hall LM, Willenborg C, Kanoni S, et al. A comprehensive 1,000 Genomes-based genome-wide association meta-analysis of coronary artery disease. Nat Genet 2015;47:1121-1130.

15. Mahajan A, Go MJ, Zhang W, Below JE, Gaulton KJ, Ferreira T, et al. Genome-wide trans-ancestry meta-analysis provides insight into the genetic architecture of type 2 diabetes susceptibility. Nat Genet 2014;46:234-244.

16. Tuma Z, Kuncova J, Mares J, Matejovic M. Mitochondrial proteomes of porcine kidney cortex and medulla: foundation for translational proteomics. Clin Exp Nephrol 2016;20:39-49.

17. Davids M, Ndika JDT, Salomons GS, Blom HJ, Teerlink T. Promiscuous activity of arginine:glycine amidinotransferase is responsible for the synthesis of the novel cardiovascular risk factor homoarginine. FEBS Lett 2012;586:3653-3657.

18. Choe C, Atzler D, Wild PS, Carter AM, Böger RH, Ojeda F, et al. Homoarginine levels are regulated by L-arginine:glycine amidinotransferase and affect stroke outcome: results from human and murine studies. Circulation 2013;128:1451-1461.

19. Rodionov RN, Oppici E, Martens-Lobenhoffer J, Jarzebska N, Brilloff S, Burdin D, et al. A Novel Pathway for Metabolism of the Cardiovascular Risk Factor Homoarginine by alanine:glyoxylate aminotransferase 2. Sci Rep 2016;6:35277.

20. Shin S, Fauman EB, Petersen A, Krumsiek J, Santos R, Huang J, et al. An atlas of genetic influences on human blood metabolites. Nat Genet 2014;46:543-550.

21. Pattaro C, Teumer A, Gorski M, Chu AY, Li M, Mijatovic V, et al. Genetic associations at 53 loci highlight cell types and biological pathways relevant for kidney function. Nat Commun 2016;7:10023.

22. Seppälä I, Kleber ME, Lyytikäinen L, Hernesniemi JA, Mäkelä K, Oksala N, et al. Genome-wide association study on dimethylarginines reveals novel AGXT2 variants associated with heart rate variability but not with overall mortality. Eur Heart J 2014;35:524-531.

*Supplementary Figures*

**Figure S1. Serum concentrations of homoarginine (hArg) (µmol/L) by the use of different hormonal contraceptive methods.** Combined oral contraceptive pills containing oestrogen (COCPs) as well as different forms of progestin-only contraceptives (POCs) including pills and intrauterine systems (IUSs). Box plots are shown as median (black horizontal line) and 25th and 75th percentiles (represented by grey boxes, interquartile range), and the whiskers (whiskers represent the highest and lowest values still within 1.5 times the interquartile range). P-values are derived from the non-parametric Kruskall-Wallis test.

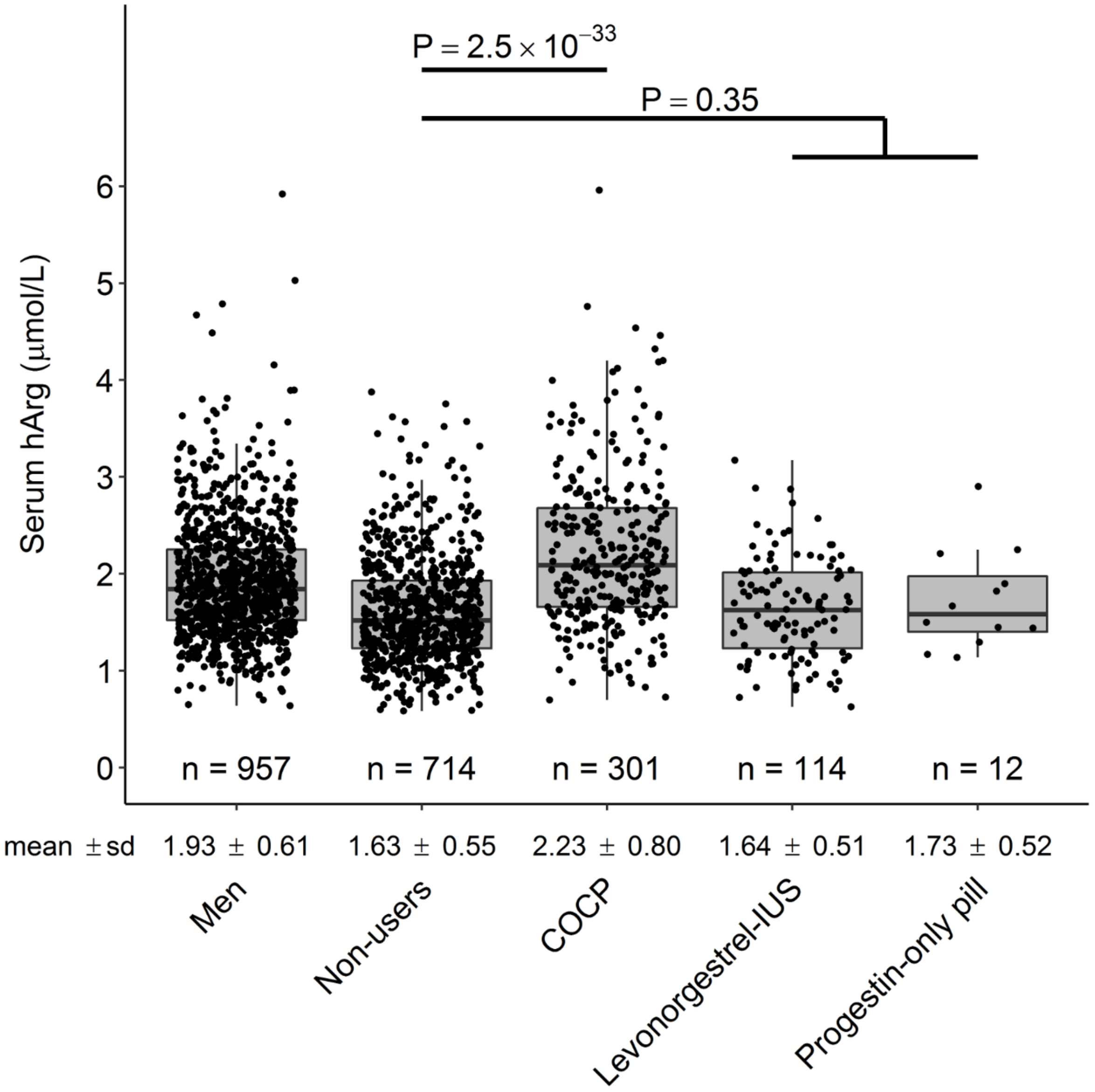

**Figure S2. Cross-sectional and longitudinal associations of baseline hArg with all 228 metabolites for both sexes separately.** Models are adjusted for age, BMI, daily smoking, serum SHBG and oral contraceptives use (women) as in **Figure 2**.

## Lipoprotein subclasses – Concentration

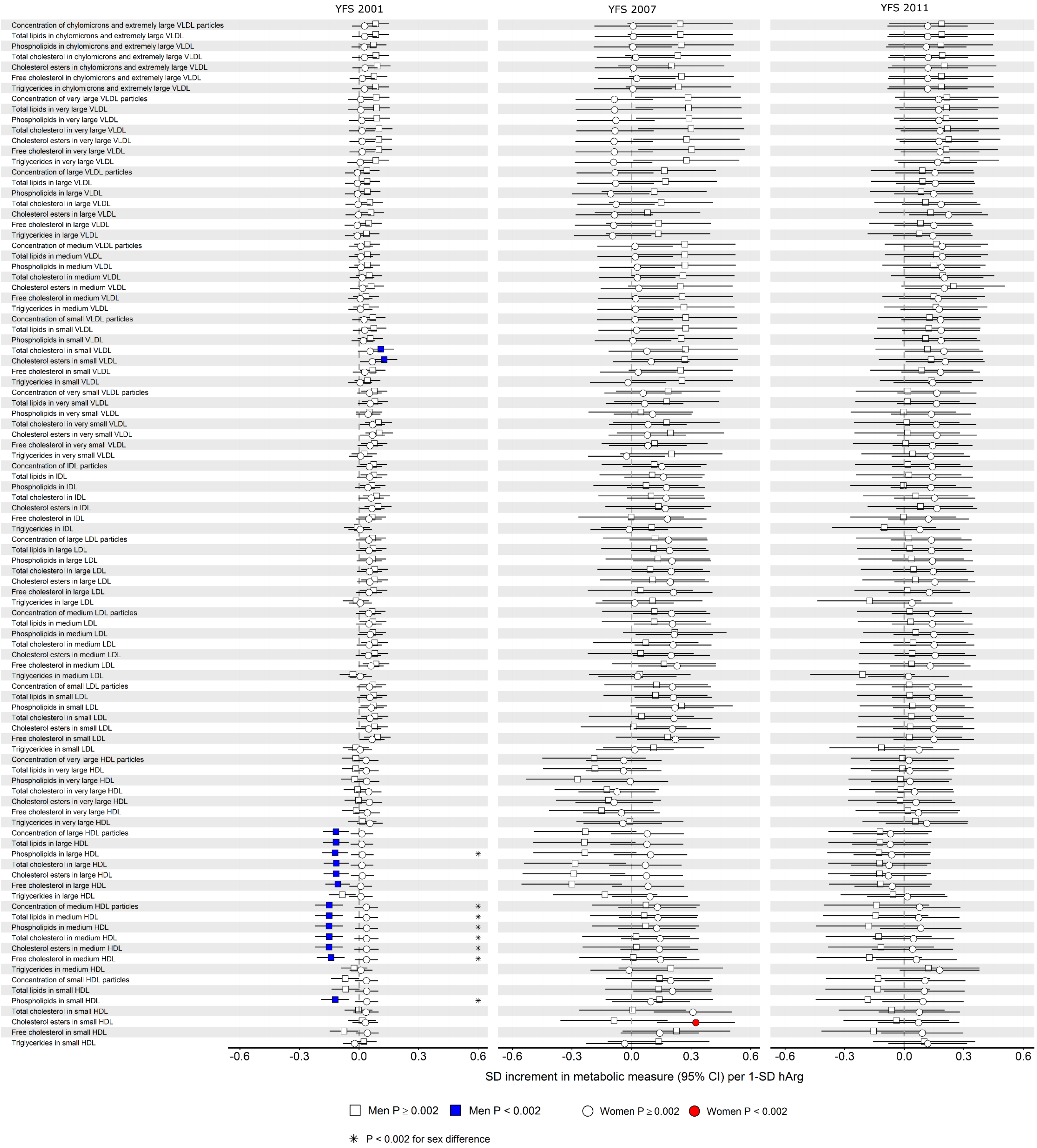

## Lipoprotein subclasses – Composition

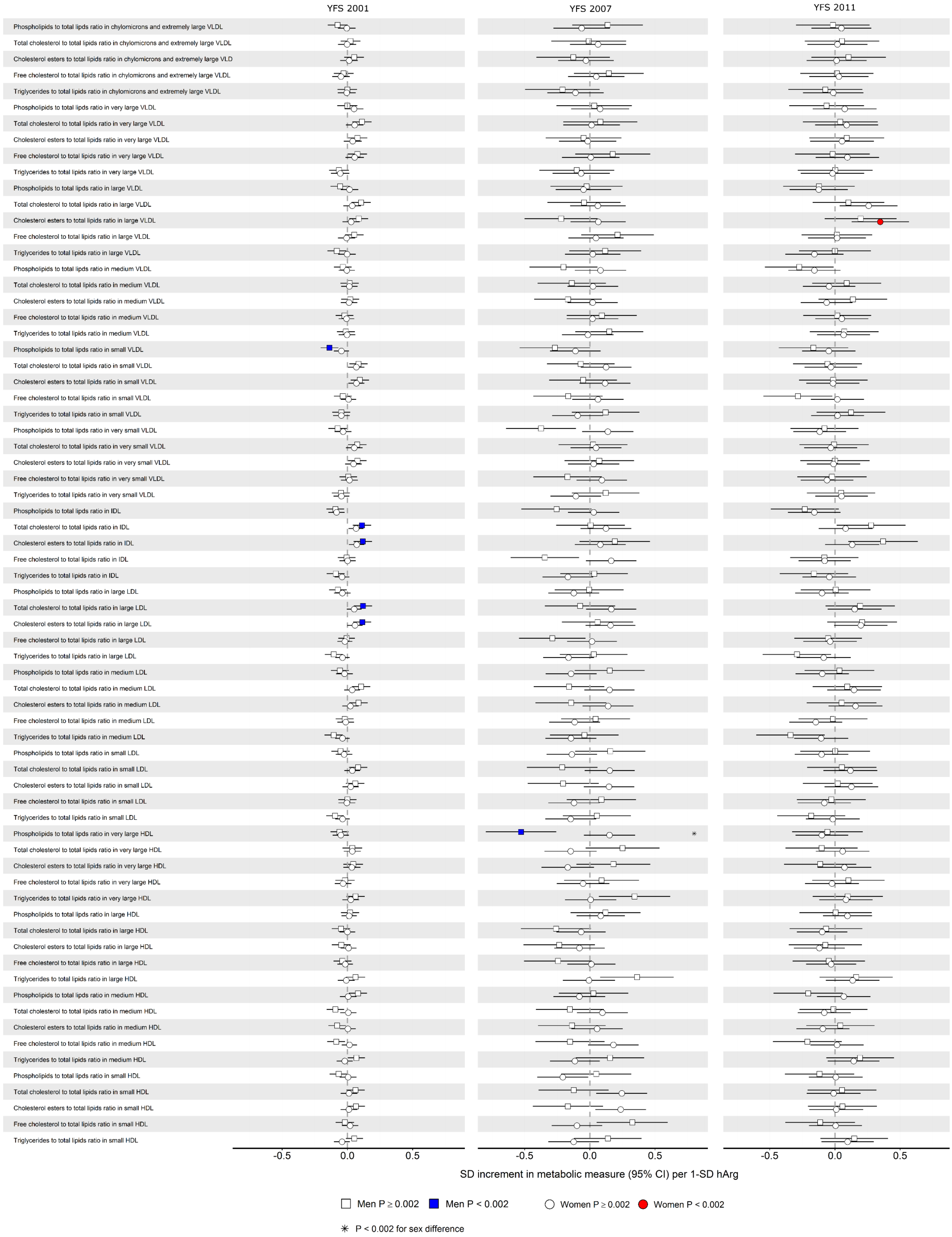

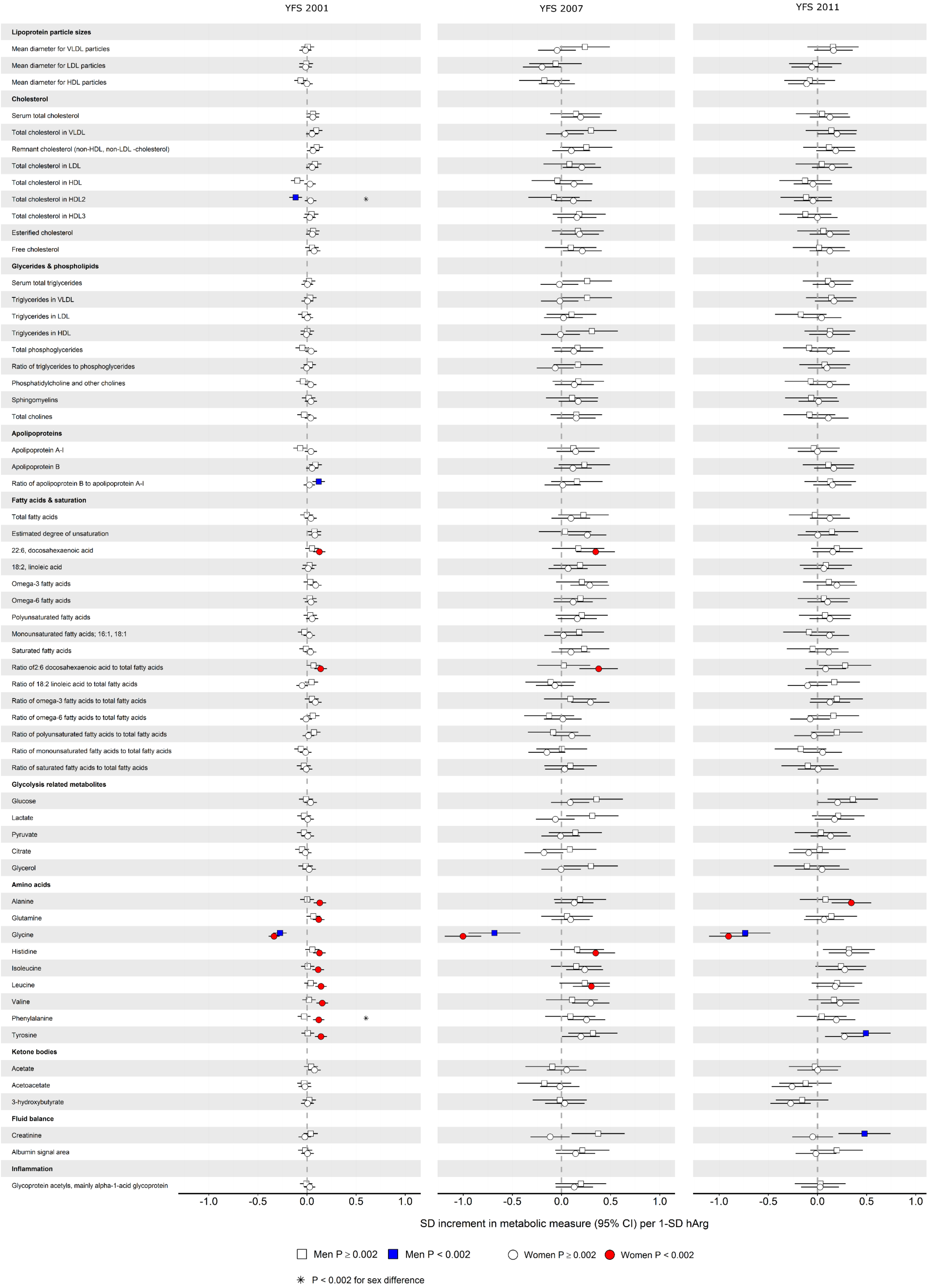

YFS 2001
YFS 2007
YFS 2011
Lipoprotein particle sizes
Mean diameter for VLDL particles
Mean diameter for LDL particles
Mean diameter for HDL particles
Cholesterol
Serum total cholesterol
Total cholesterol in VLDL
Remnant cholesterol (non-HDL, non-LDL -cholesterol)
Total cholesterol in LDL
Total cholesterol in HDL
Total cholesterol in HDL2
Total cholesterol in HDL3
Esterified cholesterol
Free cholesterol
Glycerides & phospholipids
Serum total triglycerides
Triglycerides in VLDL
Triglycerides in LDL
Triglycerides in HDL
Total phosphoglycerides
Ratio of triglycerides to phosphoglycerides
Phosphatidylcholine and other cholines
Sphingomyelins
Total cholines
Apolipoproteins
Apolipoprotein A-I
Apolipoprotein B
Ratio of apolipoprotein B to apolipoprotein A-I
Fatty acids & saturation
Total fatty acids
Estimated degree of unsaturation
22:6, docosahexaenoic acid
18:2, linoleic acid
Omega-3 fatty acids
Omega-6 fatty acids
Polyunsaturated fatty acids
Monounsaturated fatty acids; 16:1, 18:1
Saturated fatty acids
Ratio of2:6 docosahexaenoic acid to total fatty acids
Ratio of 18:2 linoleic acid to total fatty acids
Ratio of omega-3 fatty acids to total fatty acids
Ratio of omega-6 fatty acids to total fatty acids
Ratio of polyunsaturated fatty acids to total fatty acids
Ratio of monounsaturated fatty acids to total fatty acids
Ratio of saturated fatty acids to total fatty acids
Glycolysis related metabolites
Glucose
Lactate
Pyruvate
Citrate
Glycerol
Amino acids
Alanine
Glutamine
Glycine
Histidine
Isoleucine
Leucine
Valine
Phenylalanine
Tyrosine
Ketone bodies
Acetate
Acetoacetate
3-hydroxybutyrate
Fluid balance
Creatinine
Albumin signal area
Inflammation
Glycoprotein acetyls, mainly alpha-1-acid glycoprotein
-1.0
-0.5
0.0
0.5
1.0
SD increment in metabolic measure (95% CI) per 1-SD hArg
Men P ≥ 0.002
Men P < 0.002
Women P ≥ 0.002
Women P < 0.002
P < 0.002 for sex difference

**Figure S3. Cross-sectional and longitudinal associations of baseline hArg with all 228 metabolites for both sexes combined.** Models are adjusted for age, BMI, daily smoking, serum SHBG and oral contraceptives use (women) as in **Figure 2**.

### Lipoprotein subclasses – Concentration

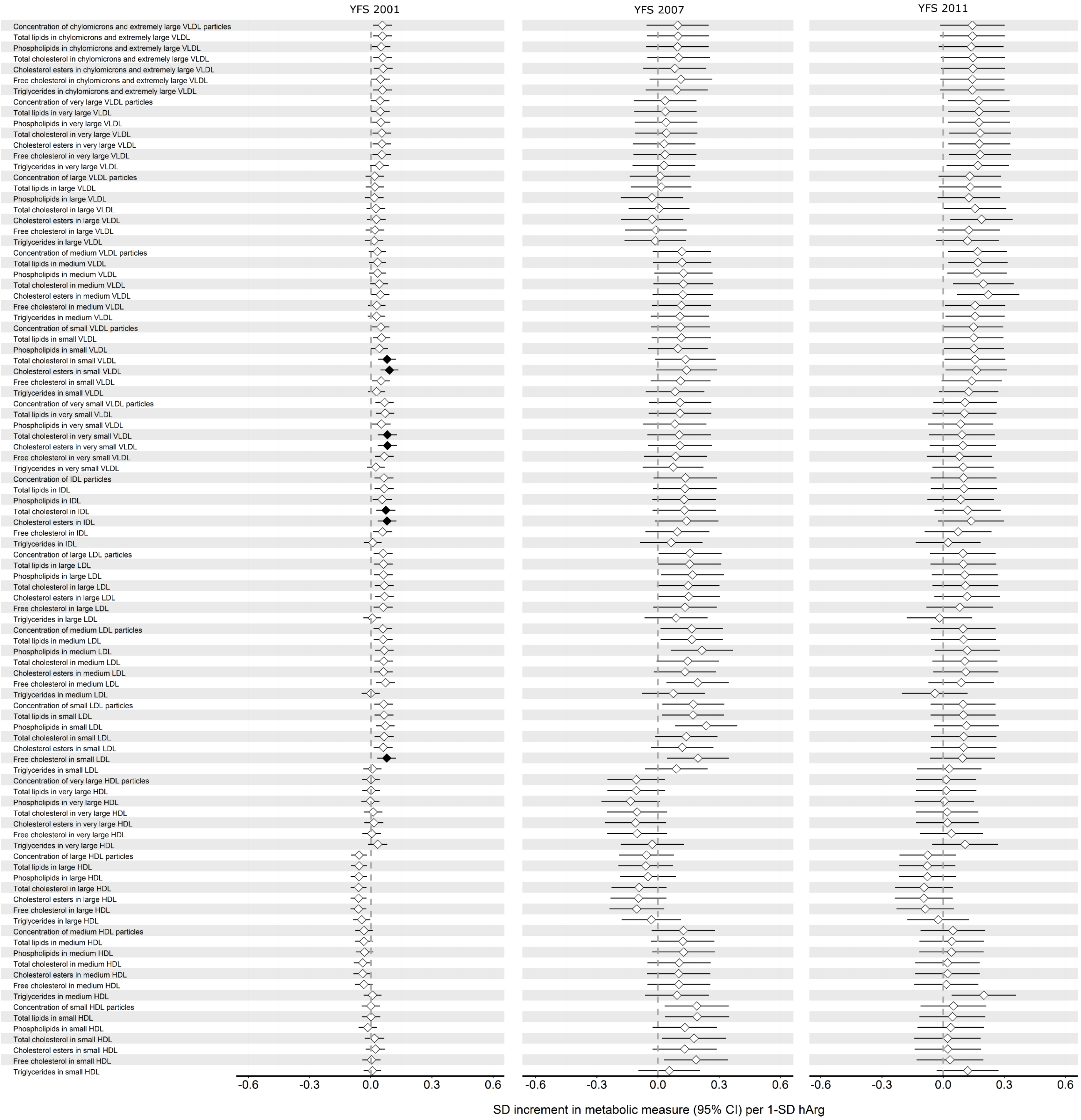

**Lipoprotein subclasses – Composition**

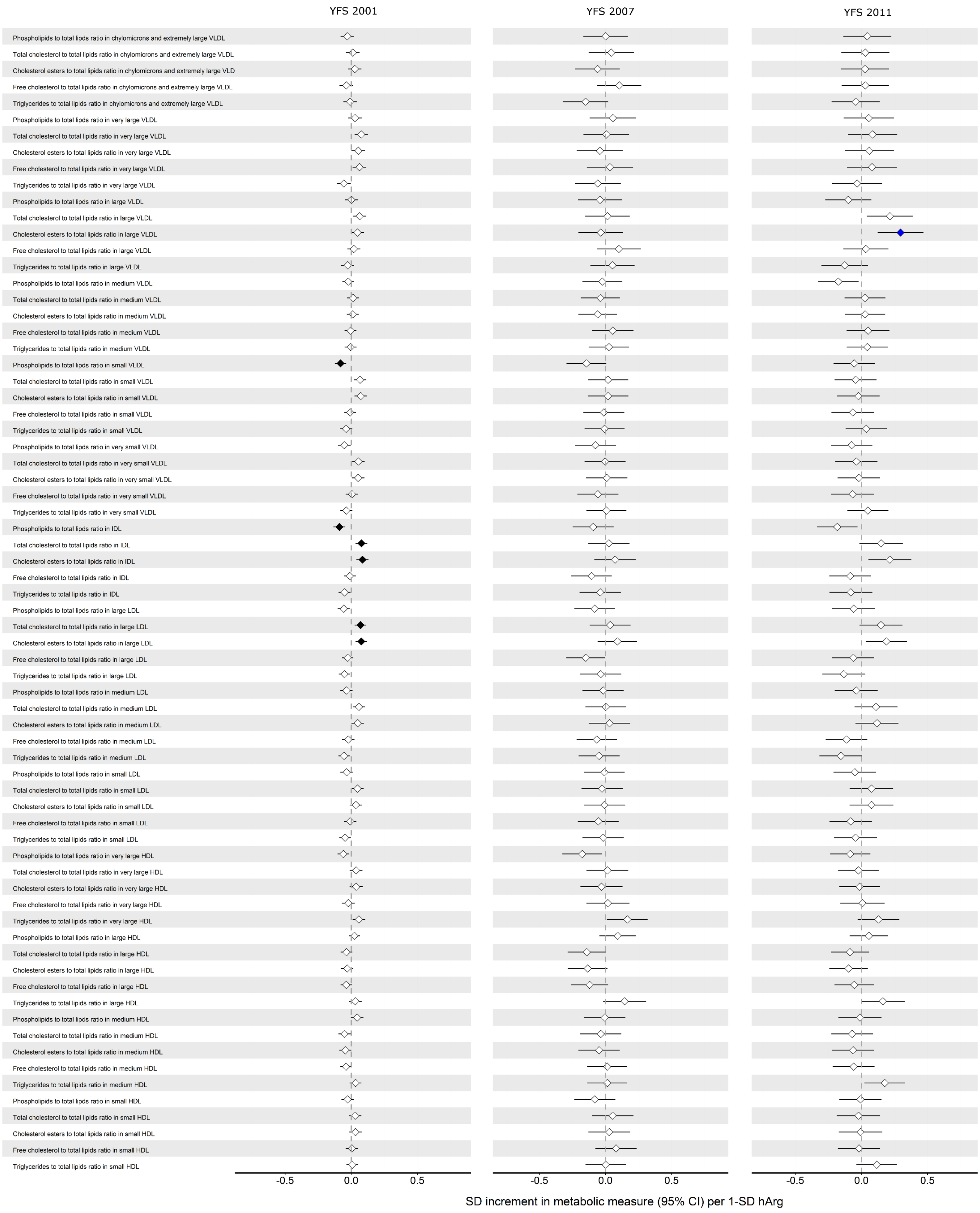

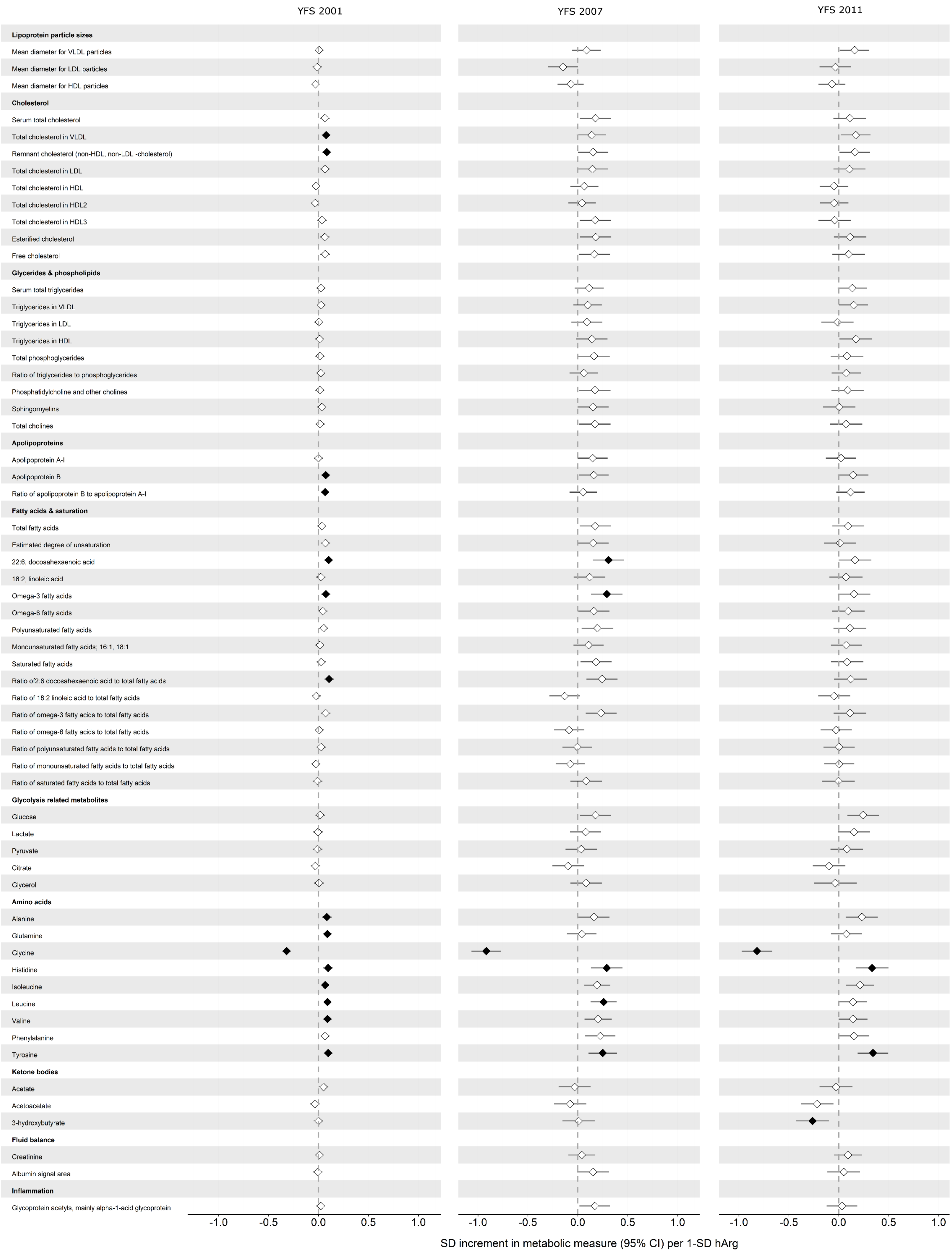

YFS 2001
YFS 2007
YFS 2011
Lipoprotein particle sizes
Mean diameter for VLDL particles
Mean diameter for LDL particles
Mean diameter for HDL particles
Cholesterol
Serum total cholesterol
Total cholesterol in VLDL
Remnant cholesterol (non-HDL, non-LDL -cholesterol)
Total cholesterol in LDL
Total cholesterol in HDL
Total cholesterol in HDL2
Total cholesterol in HDL3
Esterified cholesterol
Free cholesterol
Glycerides & phospholipids
Serum total triglycerides
Triglycerides in VLDL
Triglycerides in LDL
Triglycerides in HDL
Total phosphoglycerides
Ratio of triglycerides to phosphoglycerides
Phosphatidylcholine and other cholines
Sphingomyelins
Total cholines
Apolipoproteins
Apolipoprotein A-I
Apolipoprotein B
Ratio of apolipoprotein B to apolipoprotein A-I
Fatty acids & saturation
Total fatty acids
Estimated degree of unsaturation
22:6, docosahexaenoic acid
18:2, linoleic acid
Omega-3 fatty acids
Omega-6 fatty acids
Polyunsaturated fatty acids
Monounsaturated fatty acids; 16:1, 18:1
Saturated fatty acids
Ratio of2:6 docosahexaenoic acid to total fatty acids
Ratio of 18:2 linoleic acid to total fatty acids
Ratio of omega-3 fatty acids to total fatty acids
Ratio of omega-6 fatty acids to total fatty acids
Ratio of polyunsaturated fatty acids to total fatty acids
Ratio of monounsaturated fatty acids to total fatty acids
Ratio of saturated fatty acids to total fatty acids
Glycolysis related metabolites
Glucose
Lactate
Pyruvate
Citrate
Glycerol
Amino acids
Alanine
Glutamine
Glycine
Histidine
Isoleucine
Leucine
Valine
Phenylalanine
Tyrosine
Ketone bodies
Acetate
Acetoacetate
3-hydroxybutyrate
Fluid balance
Creatinine
Albumin signal area
Inflammation
Glycoprotein acetyls, mainly alpha-1-acid glycoprotein
-1.0 -0.5 0.0 0.5 1.0
-1.0 -0.5 0.0 0.5 1.0
-1.0 -0.5 0.0 0.5 1.0
SD increment in metabolic measure (95% CI) per 1-SD hArg

**Figure S4. Cross-sectional association of hArg with 73 metabolites adjusted for age, BMI and daily smoking.**

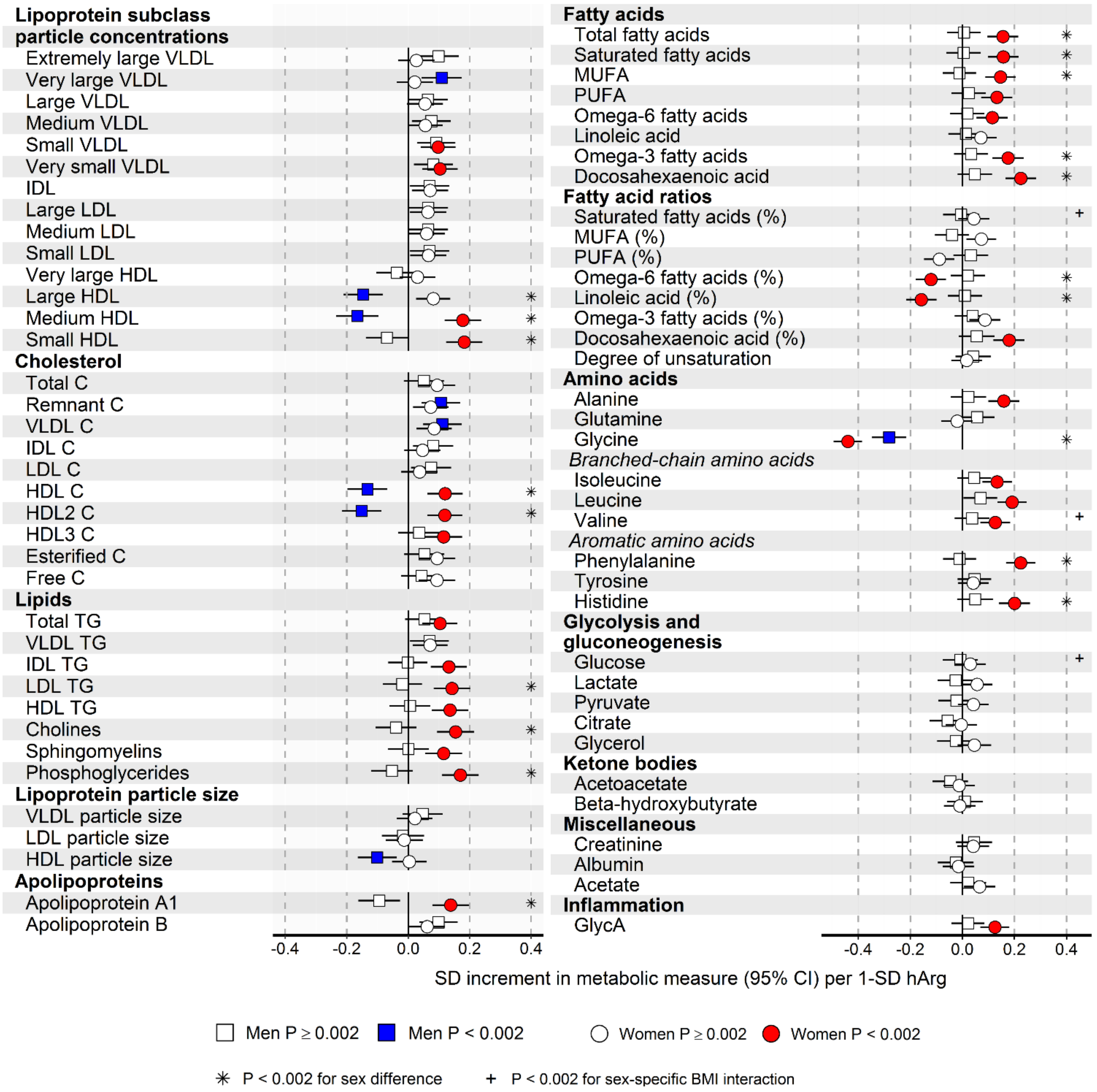

**Figure S5. Tissue-specific *GATM* mRNA expression and rs1153858.** The first boxplot (**A**) illustrates tissue-specific *GATM* mRNA expression values by sex (red, women; blue, men). Expression values are shown in log10-transformed RPKM (Reads Per Kilobase of transcript per Million mapped reads), calculated from a gene model with isoforms collapsed to a single gene. The higher the log10(RPKM) the higher the mRNA expression. Box plots are shown as median and 25th and 75th percentiles; points are displayed as outliers if they are above or below 1.5 times the interquartile range. The second boxplot (**B**) shows *GATM* mRNA expression values by the *GATM* rs1153858 genotype groups in three selected tissues (skeletal muscle, thyroid and whole blood). All illustrations are from the Genotype-Tissue Expression (GTEx) project website: http://www.gtexportal.org/**.**

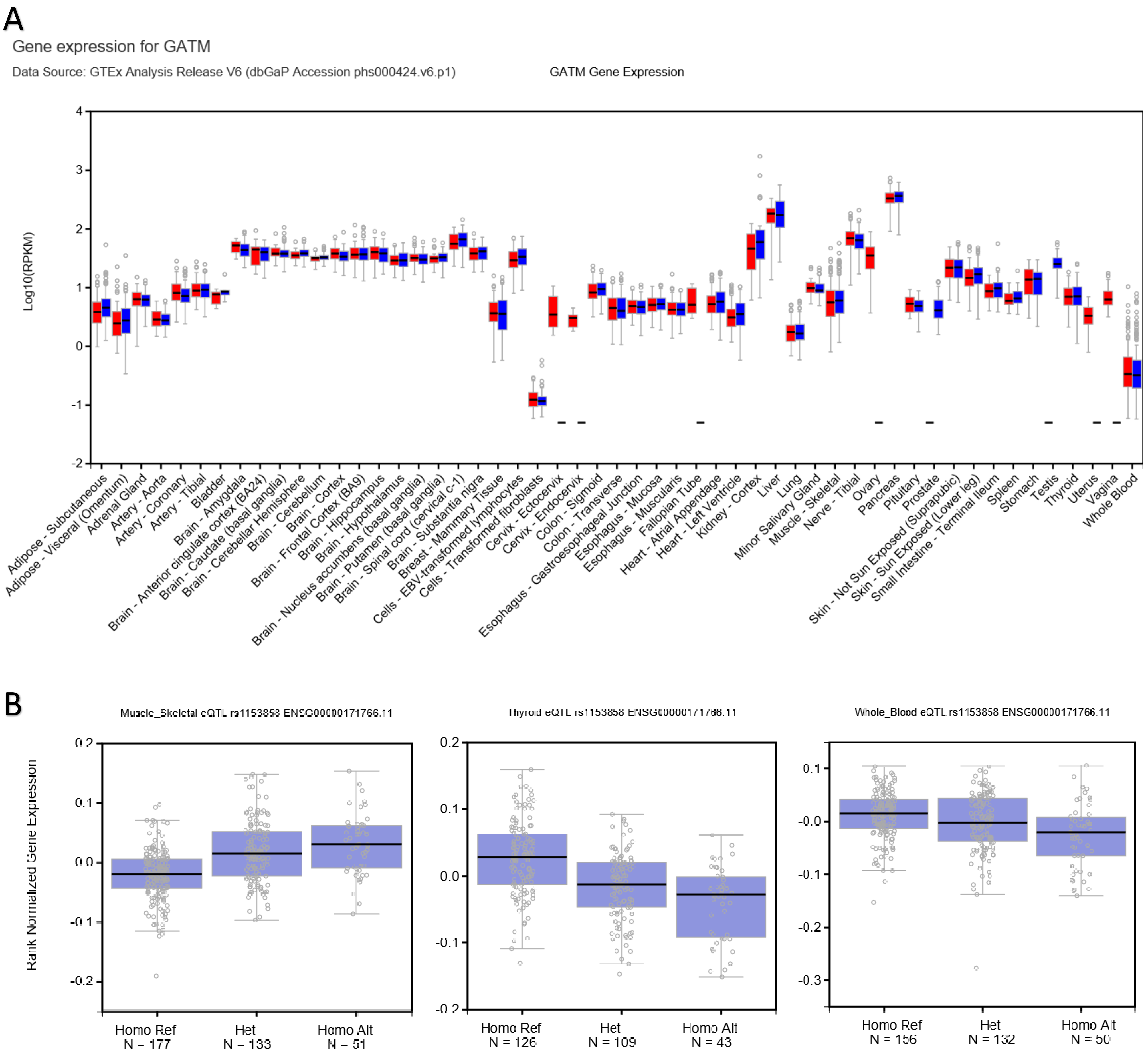

**Figure S5. Continues from the last page.** The first panel (**C**) shows the tissue-specific associations of *GATM* rs1153858 with *GATM* mRNA expression in all tissues in the GTEx project. The second panel (**D**) shows the linkage disequilibrium (LD) structure of the *GATM* locus. The *GATM* rs1153858 variant is marked with bold font. All illustrations are from the Genotype-Tissue Expression (GTEx) project website: http://www.gtexportal.org/**.**

C

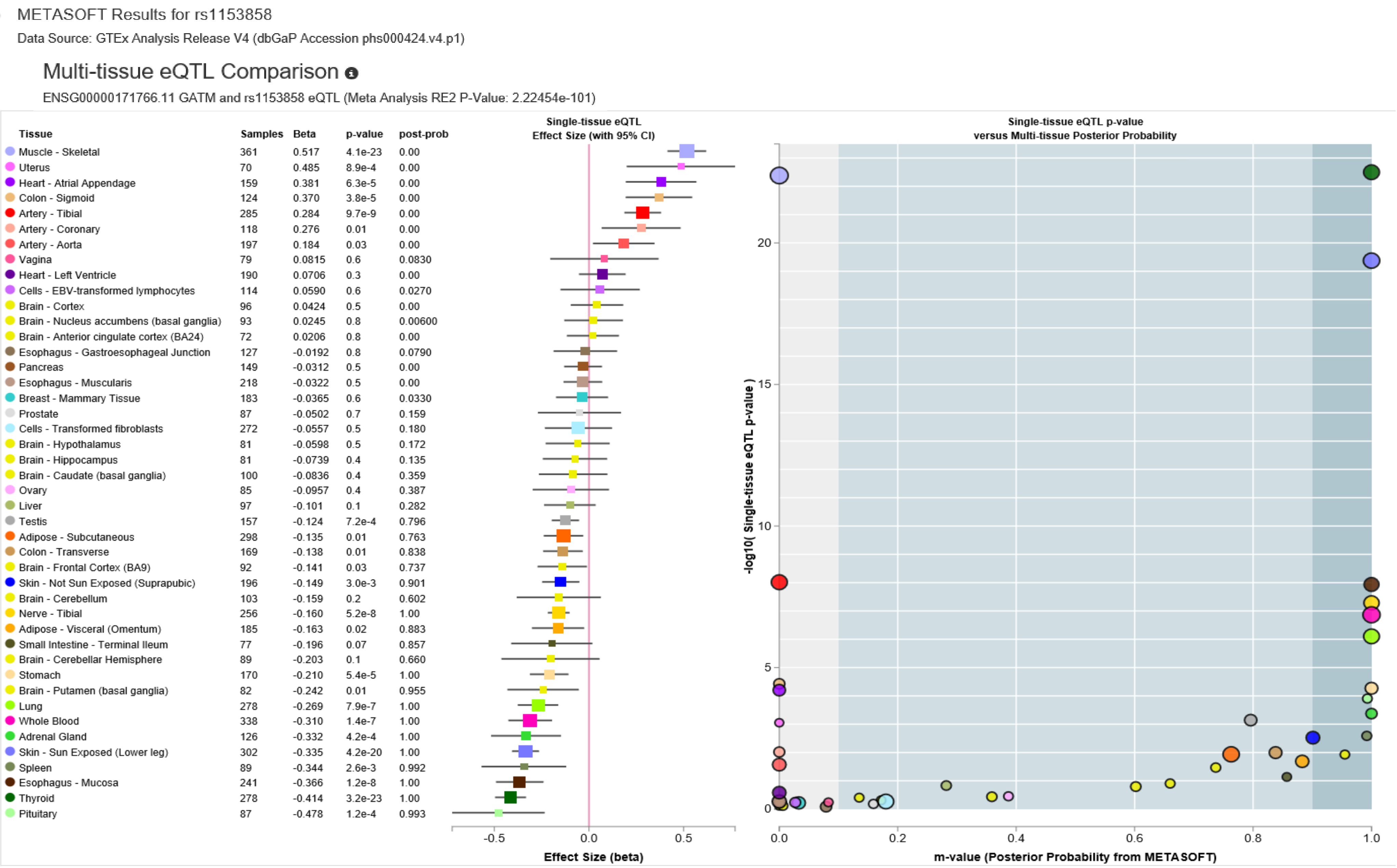


D

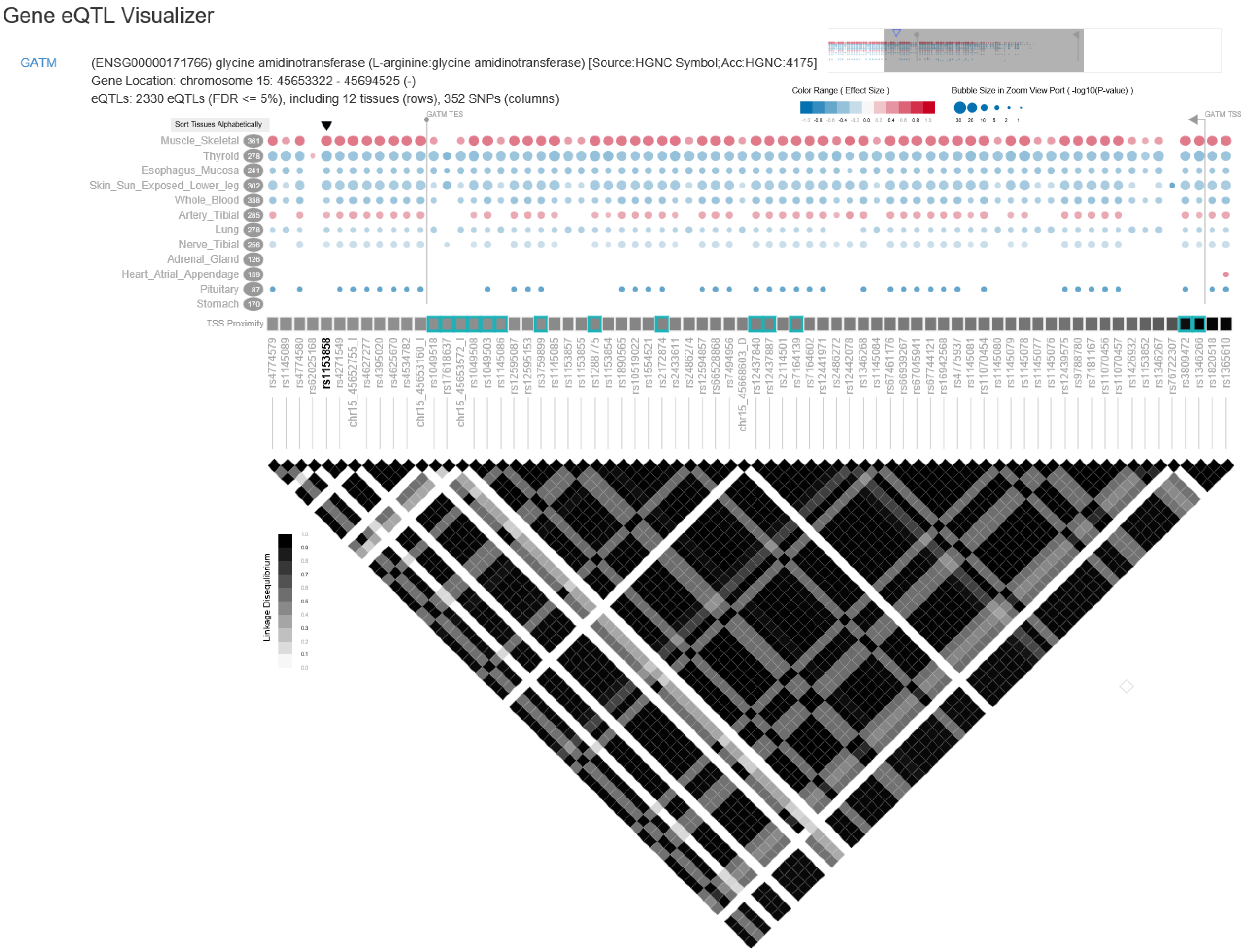

**Figure S6. A hypothetical model of hArg metabolism in humans.** A model of hArg and creatine synthesis via the AGAT enzyme (encoded by the *GATM* gene) and its intra mitochondrial substrate bioavailability based on GWAS identified single-nucleotide polymorphisms associated with circulating hArg levels [3]. Although several organs and tissues are capable of the hArg and creatine biosynthesis by AGAT, the kidneys play a pivotal role in the formation and release of hArg and the creatine precursor guanidinoacetate (GAA) into the systemic circulation. Amino acids are readily filtrated into the filtrate in the kidneys and reabsorbed in the renal cortical proximal tubules for metabolism. AGAT and AGXT2 proteins are strongly expressed in human renal tubular cells (www.proteinatlas.org) and upregulated in the porcine isolated renal cortical mitochondria compared to those isolated from the medulla [16]. AGAT catalyses the formation of GAA and ornithine from arginine and glycine as well as the formation of hArg and ornithine from arginine and lysine [17,18]. The decreased bioavailability of glycine for the GAA synthesis due to the decreased and increased activities of AGXT2 and CPS1, respectively, may shift the production of GAA by AGAT towards hArg explaining the associations of the *CPS1* and *AGXT2* missense variants with circulating hArg levels. In addition, hArg is directly metabolized to 6-guanidino-2-oxocaproic acid (GOCA) by AGXT2 [19]. The direction of association of G*ATM* rs1153858 with *GATM* mRNA expression is tissue-specific as illustrated in **Figure S5.** Directions of the effects of the *CPS1*, *AGXT2* and *GATM* variants on the enzyme activities or mRNA expression are presented by arrows. Directions and p-values of associations of the hArg associated genetic variants (marked by different colours) with blood metabolites are presented by arrows and the reference additionally marked: **↑** or **↓** = $P<5\times10^{-8}$; ↑ or ↓ = P<0.001; ↗ or ↘ = P<0.05; ↔ = P>0.05; 1 = Shin et al. [20], 2 = Kleber et al. [3], 3 = Kettunen et al. [5], 4 = Pattaro et al. [21], 5 = Seppälä et al. [22]. GATM or AGAT, glycine amidinotransferase or L-arginine:glycine amidinotransferase; AGXT2, alanine-glyoxylate aminotransferase 2; CPS1, carbamoyl-phosphate synthase 1; GCS, glycine cleavage system; GAMT, guanidinoacetate N-methyltransferase; ADMA, asymmetric dimethylarginine; SDMA, symmetric dimethylarginine; DMGV, α-keto-δ-(N,N-dimethylguanidino)valeric acid; DM'GV, α-keto-δ-(N,N'-dimethylguanidino)valeric acid.

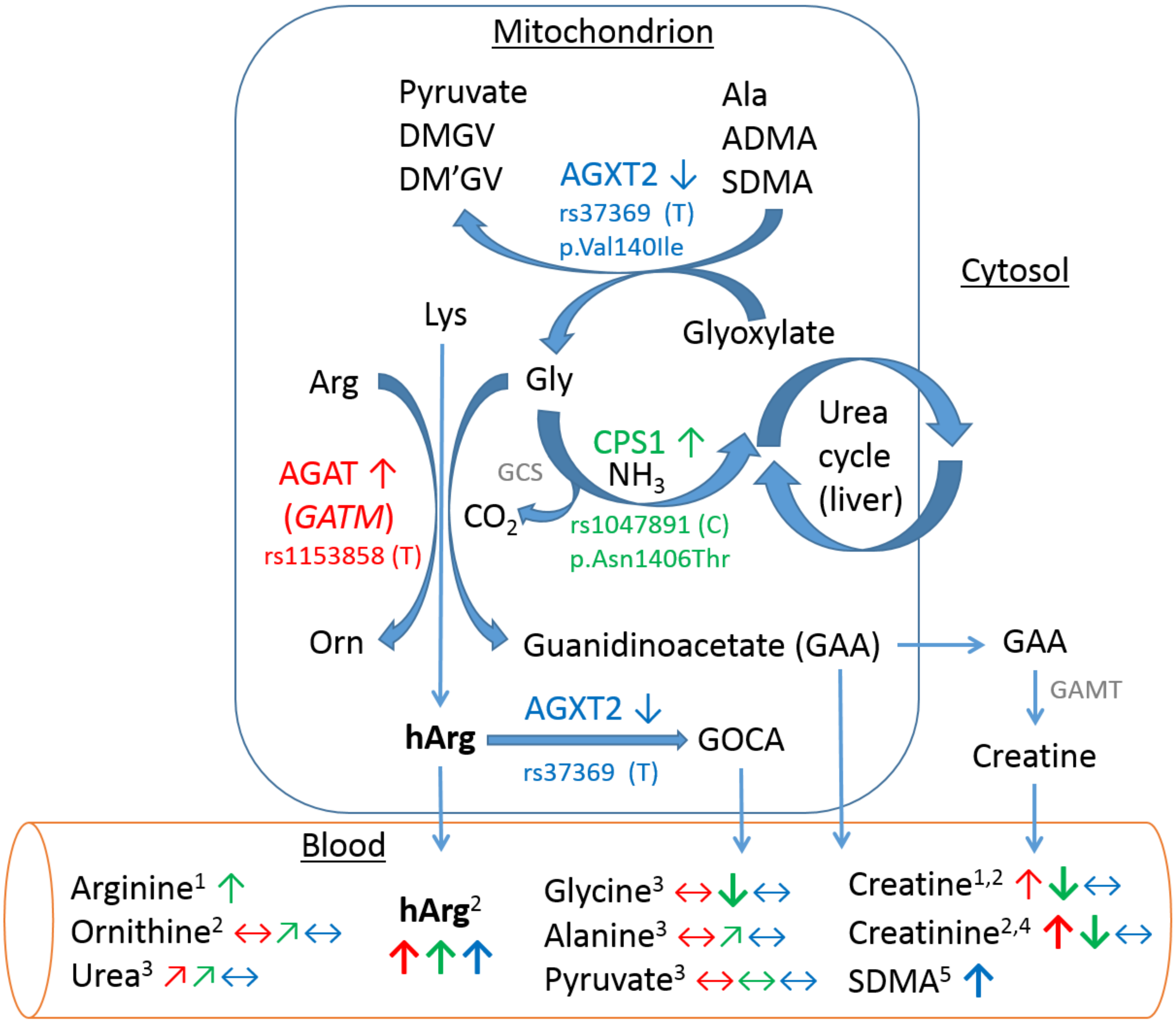

Supplement: Supplementary file 1 — Supplementary Information [file 41598_2017_1274_MOESM1_ESM.pdf]
